# Combined metabolomic and lipidomic analysis uncovers metabolic profile and biomarkers for papillary thyroid carcinoma

**DOI:** 10.1038/s41598-023-41176-4

**Published:** 2023-10-17

**Authors:** Zipeng Wang, Yiqin Yang, Yurong Xing, Dandan Si, Suhua Wang, Jiashuo Lin, Cai Li, Ji Zhang, Detao Yin

**Affiliations:** 1 Department of Thyroid Surgery, The First Affiliated Hospital of Zhengzhou University, Zhengzhou, 450052 China; 2 Engineering Research Center of Multidisciplinary Diagnosis and Treatment of Thyroid Cancer of Henan Province, Zhengzhou, 450052 China; 3 Key Medicine Laboratory of Thyroid Cancer of Henan Province, Zhengzhou, 450052 China; 4 Department of Pharmacy, The First Affiliated Hospital of Zhengzhou University, Zhengzhou, 450052 China; 5 Henan Key Laboratory of Precision Clinical Pharmacy, Zhengzhou University, Zhengzhou, 450052 China; 6 Physical Examination Center, The First Affiliated Hospital of Zhengzhou University, Zhengzhou, 450052 China; 7 SCIEX China, Beijing, 10015 China; 8 School of Medicine, Zhengzhou University, Zhengzhou, 450052 China

**Keywords:** Diagnostic markers, Cancer metabolism, Thyroid diseases

## Abstract

Papillary thyroid carcinoma (PTC) is the most common endocrine malignancy with a rapidly increasing incidence. The pathogenesis of PTC is unclear, but metabolic and lipidomic reprogramming may play a role in tumor growth. We applied ultra-performance liquid chromatography-tandem mass spectrometry to perform widely targeted metabolomics and lipidomics on plasma samples from 94 patients with PTC and 100 healthy controls. We identified 113 differential metabolites and 236 differential lipids, mainly involved in branched-chain amino acid metabolism, glutamate and glutamine metabolism, tricarboxylic acid cycle, and lipid metabolism. We also screened three potential metabolite biomarkers: sebacic acid, L-glutamine, and indole-3-carboxaldehyde. These biomarkers showed excellent diagnostic performance for PTC in both discovery and validation cohorts, with areas under the receiver operating characteristic curves of 0.994 and 0.925, respectively. Our findings reveal distinct metabolic and lipidomic features of PTC and provide novel targets for diagnosis and treatment.

## Introduction

Recently, the incidence of papillary thyroid carcinoma (PTC), the most common endocrine malignancy, has been rapidly increasing^1^. Early diagnosis and treatment of PTC are efficient strategies for improving the prognosis of patients with PTC^2^. Although molecular markers are valuable in diagnosing PTC, they lack specificity or have a limited positive predictive value^3^. Therefore, exploration of the pathogenesis and diagnostic biomarkers of PTC is urgently needed.

Circulating metabolic biomarkers support the understanding of tumor biology, and early diagnosis with minimum invasion. Tumor cells are highly metabolically active and undergo many metabolic reprogramming to sustain faster proliferation^4^. Metabolic reprogramming is an important feature of tumors^5^. Even when sufficient oxygen is present, tumor tissue needs to consume large amounts of glucose through glycolysis, this is called the Warburg effect. Glucose-related metabolites are substantially altered in PTC^6^ and are associated with its stemness and aggressiveness^7^. Amino acids are a resource for protein synthesis and are involved in the biosynthesis of other macromolecules. Metabolic reprogramming of amino acids promotes tumor proliferation and metastasis^8^. Disturbances in the metabolism of some amino acids in PTC have also been observed, but the metabolic pattern of amino acids in PTC remains unclear^2^. The relative concentration of branched-chain amino acids in saliva was remarkably decreased in PTC^2^. Lipids and their metabolites are used in cell membrane formation, signaling, and energy storage in normal cells, and associated with carcinogenic pathways^9^. Lipid metabolism is reprogrammed in tumors, and the perturbation of blood lipids has been identified as a risk factor for tumorigenesis^10,11^. Therefore, we speculated that metabolic reprogramming is an important feature of PTC and provides diagnostic biomarkers for PTC. Although the metabolomics and lipidomics for PTC have been studied^3^, the metabolic reprogramming characteristics of PTC have not been fully elucidated and are rarely used for PTC diagnosis.

In this study, the widely targeted metabolomics and lipidomics methods were performed to explore the metabolic reprogramming and potential metabolite biomarkers of PTC and provide potential targets for its comprehensive treatment.

## Materials and methods

### Participants and study design

The study was approved by the Ethics Committee of Scientific Research and Clinical Trial of the First Affiliated Hospital of Zhengzhou University. The ethics review approval ID was “2021-KY-1011-002”. All methods were performed in accordance with the relevant guidelines and regulations. Informed consent was obtained from all participants, and fasting whole preoperative blood samples were collected. A total of 106 preoperative blood samples from patients with PTC were collected the day before surgery from the Department of Thyroid Surgery, the First Affiliated Hospital of Zhengzhou University, between September 2021 and May 2022. The inclusion criteria were as follows: (1) patients who underwent thyroid surgery for the first time, (2) patients pathologically diagnosed with PTC, and (3) patients willing to sign an informed consent. Patients with other malignancies, thyroid dysfunction (hyperthyroidism or hypothyroidism), or two or more thyroidectomies were excluded from this study. After exclusion, 94 patients with PTC were included. In addition, 100 healthy controls (HC) were recruited from a population receiving routine physical examinations at the First Affiliated Hospital of Zhengzhou University. The inclusion criteria for HC included: (1) age- and sex-matched to the PTC group, (2) no other diseases, including liver or renal failure, and multiple organ failure combined; and (3) subjects willing to sign an informed consent. The enrollment flowchart of the participants is shown in Fig. S1.

### Reagents and sample preparation

The reagents and sample preparation were provided in the supporting information.

### Targeted metabolomic and lipidomic analysis

Widely targeted metabolomics and lipidomics were performed using a QTrap 6500 triple quadrupole linear ion-trap mass spectrometer. Multiple reaction monitoring was performed in pneumatic-assisted electrospray ionization mode while monitoring positive and negative ions. Data collection was performed using Analyst 1.7.1 software (AB Sciex, USA)^12^.

### Data analysis

The raw data files were pre-processed using MultiQuant 3.0.2 (AB Sciex, USA) software for retention time (RT) correction, peak identification, and peak integration. An appropriate internal standard (IS) for each compound was selected for peak area calibration according to the principles of similar chemical structures and RT. SIMCA14.1 software was used for principal component analysis (PCA), and the orthogonal partial least squares discriminant analysis (OPLS-DA) method was used to construct the model. Two hundred permutation tests were performed to assess whether the model overfitted. The variable importance in the projection (VIP) in the OPLS-DA model was used to identify and differentiate two groups of compounds with the highest contribution. Differential compounds were defined as *p* < 0.05, fold change (FC) ≥ 1.2 or ≤ 0.83, and VIP > 1.

Heatmaps were drawn based on R 4.1.3. The pheatmap package was used for heatmaps. Multivariate logistic regression was constructed using stepwise regression based on the differential compounds, and receiver operating characteristic (ROC) curves were established using R 4.1.3. The pROC, rms, and readr packages were used for the multivariate logistic regression and construction of the ROC curve. In the discovery cohort, differential compounds were screened, and the model was built and validated in the validation cohort. The differential compounds were imported into MetaboAnalyst (http://www.metaboanalyst.ca/) and compared with those in the Kyoto Encyclopedia of Genes and Genomes (KEGG) database for enrichment and pathway analyses.

## Results

### Patient characteristics and study design

The baseline characteristics of patients with PTC and HC were collected in Table 1. No statistical difference was detected in the demographic characteristics and laboratory test results between the PTC and HC groups (Table 1). To build a diagnostic model, both PTCs and HCs were randomly divided into discovery and validation cohorts in a 7:3 ratio. In the discovery cohort, 65 PTCs and 70 HCs were enrolled, and differential metabolites were screened and applied for diagnosis model construction. In a validation cohort of 29 PTCs and 30 HCs, we validated the screened differential metabolites and established a model to test the discriminative performance of the model. The American Joint Committee on Cancer (AJCC) TNM staging system is the most widely used and accepted cancer staging system internationally and is the standard method for staging malignant tumors in clinical practice. The T refers to the primary tumor, N refers to regional lymph node involvement, and M refers to distant metastasis. The TNM three indicators are combined to determine the stage of cancer. The TNM stage of the patients was classified according to the eighth edition of the AJCC on cancer tumor staging system. The T and N stages of PTC were not substantially different between the discovery and validation cohorts (Table 1).

**Table 1:** Clinical characteristics and laboratory data of the study population.

	Discovery cohort (n = 135)			Validation cohort (n = 59)		
	PTC (n = 65)	HC (n = 70)	*p*	PTC (n = 29)	HC (n = 30)	*p*
Age (years)	41 ± 10	42 ± 10	0.9347	40 ± 10	45 ± 11	0.1333
Male, n (%)	12 (18.5)	10 (14.3)	0.6419	4 (13.8)	8 (26.7)	0.3334
BMI (kg/m^2^)	24 ± 3	24 ± 4	0.345	24 ± 3	24 ± 3	0.9347
Hb (g/L)	130 ± 16	132 ± 15	0.4406	129 ± 10	135 ± 16	0.1433
PLT (10^9^/L)	251 ± 66	248 ± 60	0.8404	236 ± 56	225 ± 54	0.4661
Crea (μmol/L)	64 ± 15	62 ± 12	0.4025	62 ± 13	68 ± 10	0.0558
ALT (U/L)	20 ± 15	20 ± 18	0.9846	24 ± 21	18 ± 12	0.2268
AST (U/L)	19 ± 7	20 ± 9	0.2926	22 ± 12	20 ± 6	0.4697
ALP (U/L)	71 ± 20	74 ± 24	0.416	72 ± 17	74 ± 22	0.7366
T stage of PTC ^a^						
T 1	57 (87.6%)			21 (72.4%)		
T 2	8 (12.3%)			8 (27.6%)		
T 3	0			0		
T 4	0			0		
N stage^b^						
N 0	37 (56.9%)			15 (51.7%)		
N 1	28 (43.1%)			14 (48.3%)		
M stage						
M 0	65 (100.0%)			29 (100.0%)		
M 1	0			0		

Baseline characteristics of enrolled patients with PTC and healthy controls represented as mean ± standard deviation (SD) or percentage (%). *BMI* body mass index, *Hb* hemoglobin, *PLT* platelet, *Crea* creatinine, *ALT* alanine aminotransferase, *AST* aspartate aminotransferase, *ALP* alkaline phosphatase. No statistically significant difference was detected in the T-stage ^a^ (*p* = 0.069) and the N-stage ^b^ (*p* = 0.64) of PTC between the discovery and the validation groups.

### Patients with PTC show plasma metabolome alterations

In an unsupervised multivariate PCA, a separation trend was observed in plasma metabolic phenotypes between PTC and HC (Fig. S2A and D) in the discovery cohort. In the OPLS-DA, clear differences were obtained for PTC versus HC, with cumulative R^2^X = 0.41, R^2^Y = 0.87, and Q^2^ = 0.771 on the C18 column and R^2^X = 0.384, R^2^Y = 0.898, and Q^2^ = 0.79 on the HILIC column (Fig. S2B and E). The permutation test results showed negative Q^2^Y intercept, and the points of Q^2^ were lower than those of R^2^, indicating that the model was stable without overfitting (Fig. S2C and F). Similar patterns between HC and PTC were observed in the plasma samples of the validation cohort (Fig. S3).

**Figure 1:**
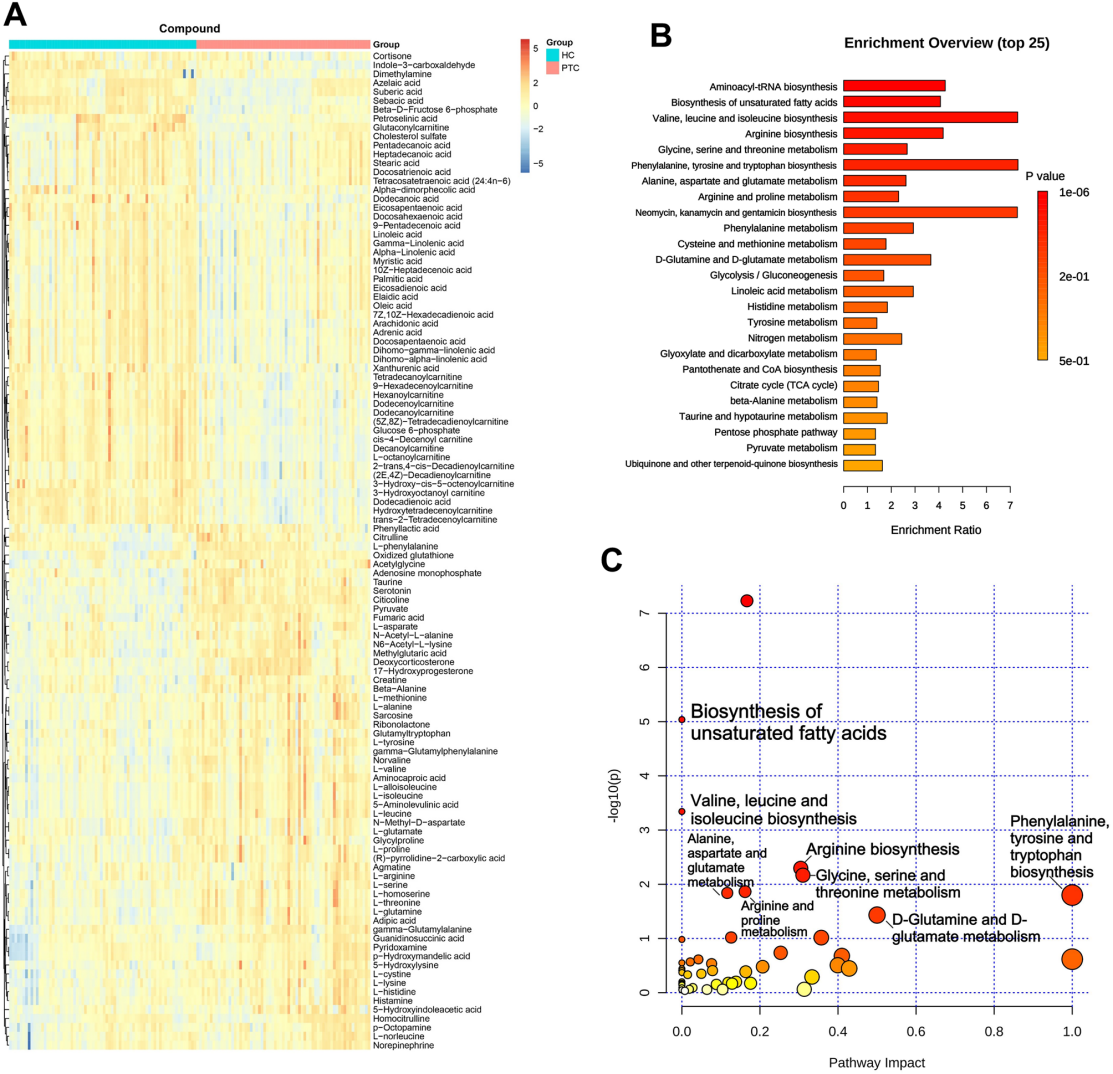
Metabolomic profiling in patients with PTC compared to that of HC. C18 and hydrophilic interaction liquid chromatography (HILIC) column differential metabolite screening between PTC and HC, enrichment analysis, and pathway analysis. Cluster heat map analysis of differential metabolites (**A**). Orange blocks represent higher relative concentrations of metabolites, and the darker the color, the higher the relative concentration. The blue color block represents the lower relative concentration of the metabolite, and the darker the color, the lower the relative concentration. Enrichment analysis of different metabolites between PTC and HC (**B**), the abscissa represents the Enrichment Ratio corresponding to each pathway, and the ordinate is the pathway name. Pathway analysis (**C**) of PTC, the color and size of each circle is based on pathway impact values (the larger the circle the higher the impact score) and *p*-values (yellow: higher *p*-values and red: lower *p*-values), respectively (**E**). *PTC* papillary thyroid carcinoma, *HC* healthy controls.

The VIP value obtained in the OPLS-DA model was used to identify the metabolites with higher contribution to the differentiation between PTC and HC groups. Differential metabolites were defined as a *p*-value < 0.05, FC ≥ 1.2 or ≤ 0.83, and VIP > 1 between PTC and HC. The differential metabolites screened by the C18 and HILIC columns were combined, and a total of 112 differential metabolites were identified based on the above filter criteria, with 59 elevated and 53 decreased metabolites in the plasma of patients with PTC (Table S1). Heatmap analysis was applied to perform hierarchical clustering of differential metabolites between the PTC and HC groups (Fig. 1A). The results showed that patients with PTC had markedly different metabolic patterns than those of healthy subjects.

**Figure 2:**
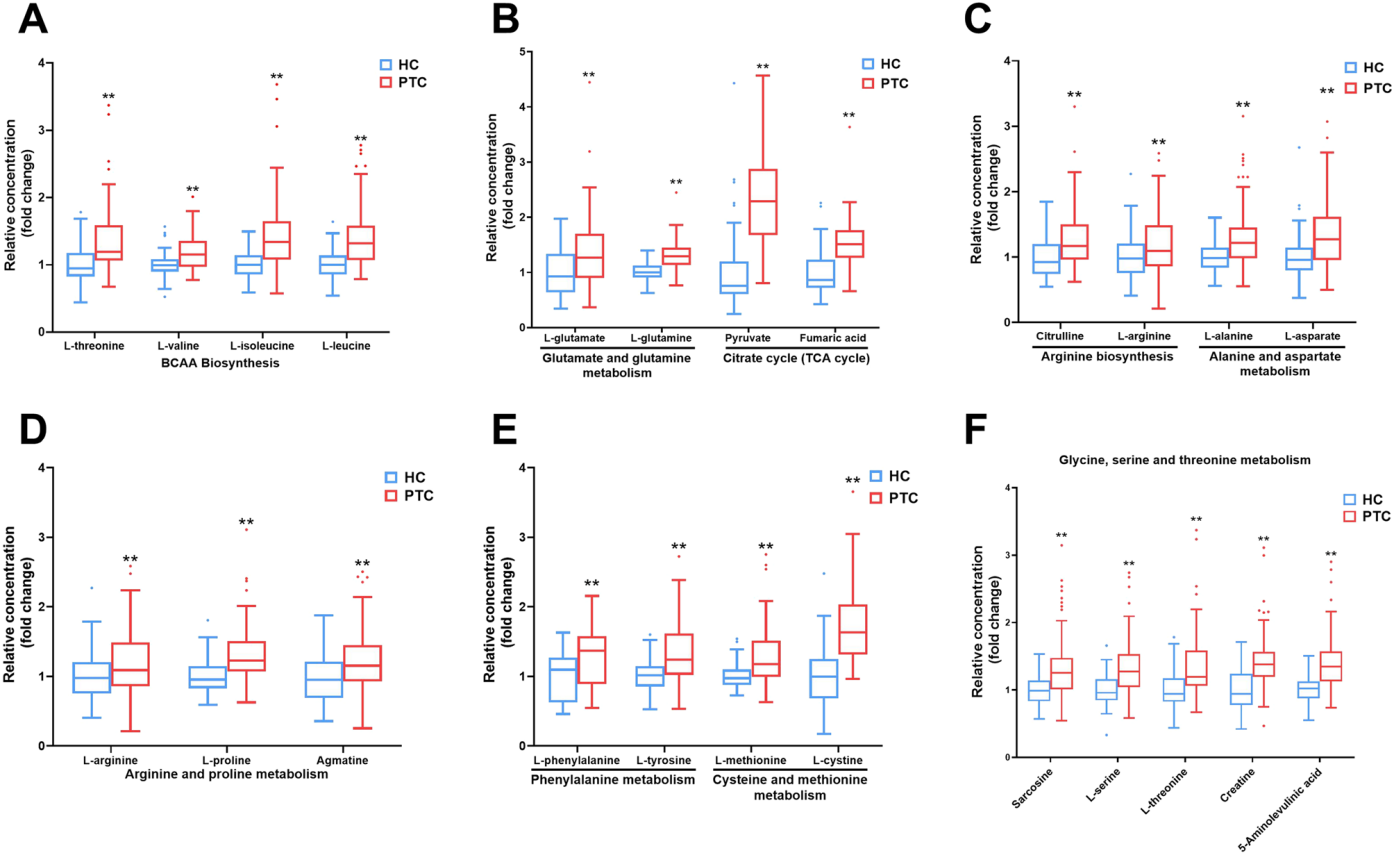
Distinct metabolic pathway disturbance in the plasma of patients with PTC. The altered metabolic pathways and relative concentrations of differential metabolites are shown by the box and whisker plots. The box plots are visualized as mean values, 25th and 75th percentiles. Whiskers denote the data outside the 25th to 75th percentile range but are not considered to be outliers. The outliers are visualized by the separate dots beyond the whiskers. *p* < 0.05 was considered significant. *p < 0.05 and **p < 0.01. BCAA (branched-chain amino acid) biosynthesis (**A**), glutamate and glutamine metabolism, TCA cycle (tricarboxylic acid cycle) (**B**), arginine biosynthesis, alanine and aspartate metabolism (**C**), arginine and proline metabolism (**D**), phenylalanine metabolism, cysteine and methionine metabolism (**E**), glycine, serine and threonine metabolism (**F**) were elevated in PTC.

MetaboAnalyst was used to assess the metabolic enrichment and pathway analysis of differential metabolites. The disordered metabolic pathways are shown in Figs. 1B and C and Table S2. Compared with those of healthy controls, the altered metabolic pathways mainly included biosynthesis of unsaturated fatty acids, as well as valine, leucine, isoleucine, arginine, glycine, serine, threonine, alanine, aspartate, glutamate, phenylalanine, tyrosine, tryptophan, glutamine, and glutamate. Moreover, patients with PTC had higher concentrations of metabolites related to branched-chain amino acids (BCAAs) biosynthesis, including L-threonine, L-valine, L-leucine, and L-isoleucine (Fig. 2A). In addition, several metabolites in the tricarboxylic acid cycle (TCA cycle) and glutamate metabolism were accumulated in the plasma of patients with PTC, such as L-glutamine, L-glutamate, pyruvate, and fumaric acid (Fig. 2B). Furthermore, increased plasma levels of other amino acids related to phenylalanine, alanine, aspartate, arginine, and proline metabolism have been observed in PTC. These data indicate that the biosynthetic and/or metabolic processes of amino acids and fatty acids (FA) are extensively altered in patients with PTC, which may contribute to disease pathogenesis and provide diagnostic biomarkers.

### PTC extensively altered lipid metabolism

Plasma lipidomic phenotypes in PTC and HC were investigated using the targeted lipidomic method, which detected over 700 lipid species belonging to 12 lipid classes. PCA showed no substantial difference in lipidomic characteristics between PTC and HC (Fig. S4A). However, the lipidomic profiles of PTC and HC were clearly distinguished in OPLS-DA, with cumulative R^2^X at 0.503, R^2^Y at 0.795, and Q^2^ at 0.492 (Fig. S4B). The permutation test of the OPLS-DA model indicated that it was stable without overfitting (Fig. S4C) in the discovery cohort. In the validation cohort, similar patterns between HC and PTC were observed in the plasma samples (Fig. S5). Differential lipids were defined as *p* < 0.05, FC ≥ 1.2 or ≤ 0.83, and VIP > 1 between PTC and HC. A total of 236 differential lipids were identified between both groups, among which 207 were elevated and 29 were decreased in the plasma of patients with PTC (Table S3). Cluster heatmap analysis also demonstrated that the PTC and HC groups had substantially different lipid patterns (Fig. S4D).

**Figure 3:**
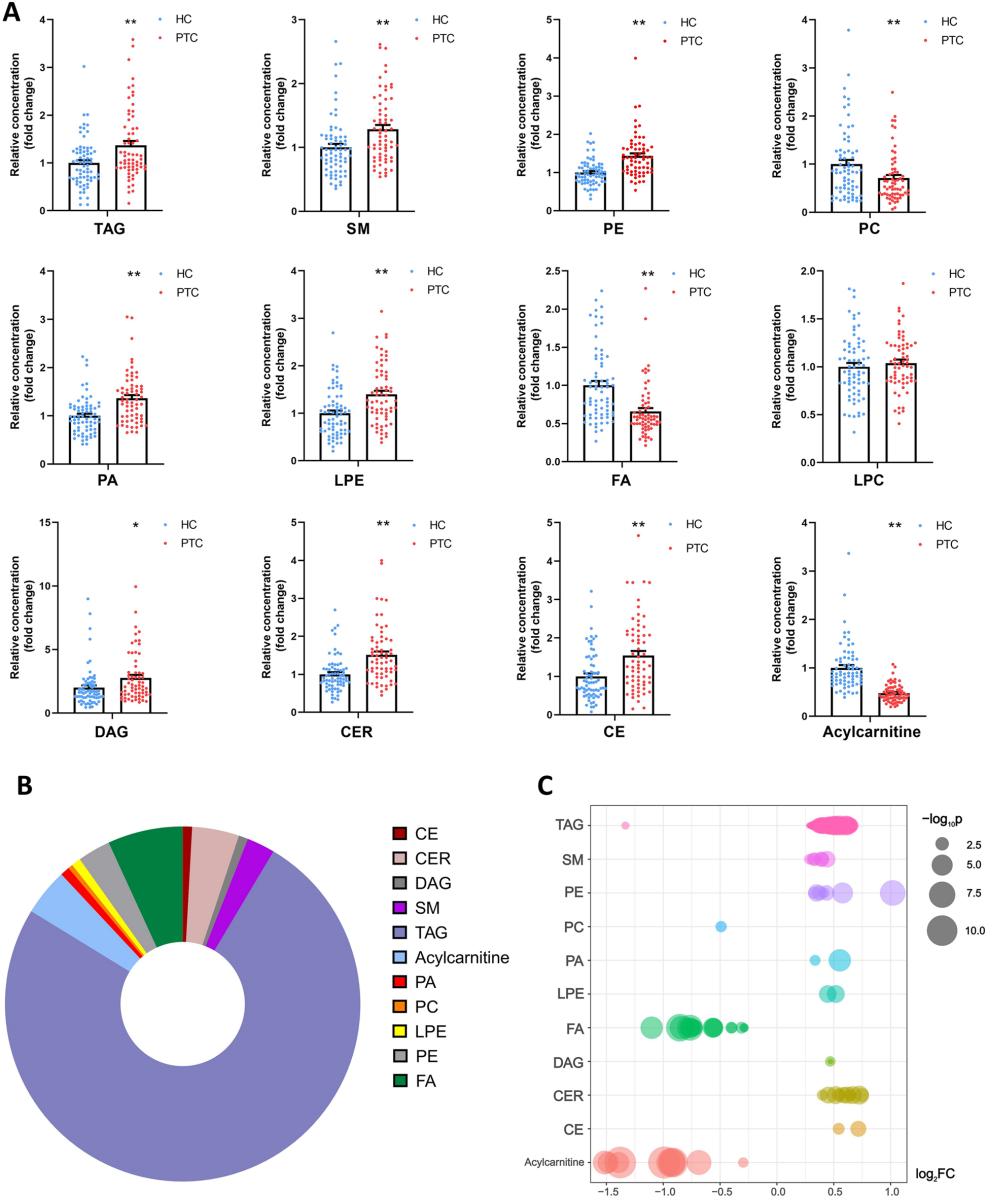
Perturbation of plasma lipids in the plasma of patients with PTC. Histogram of the relative concentration of plasma lipids in PTC and HC, and each point represents a sample (**A**). All results are represented as mean ± SEM, with *p < 0.05 and **p < 0.01 indicating significant differences between both groups. The sector graph (**B**) reflects the distribution of the differential lipid class. In the bubble chart (**C**), the larger the point, the lower the *p*-value. -log_10_p was mapped to points of different sizes. The ordinate represents the name of each differential lipid class, and each point represents a lipid. The abscissa is the log_2_FC value. Triacylglyceride (TAG), sphingomyelin (SM), phosphatidyl ethanolamine (PE), phosphatidic acid (PA), lysophosphatidic ethanolamine (LPE), diacylglycerol (DAG), ceramide (CER), and cholesteryl ester (CE) were increased, whereas phosphatidylcholine (PC), fatty acid (FA), and acylcarnitine were decreased in the PTC group.

Figure 3A shows the relative concentrations of lipids in PTC and HC samples. Figure 3B shows that the percentage of differential lipids, 75.21%, 6.84%, 4.27%, and 4.27% of the differential lipids in PTC belong to the lipid classes of triacylglyceride (TAG), FA, acylcarnitine, and ceramide (CER), respectively. In particular, the total contents of TAG, sphingomyelin (SM), phosphatidyl ethanolamine (PE), phosphatidic acid (PA), lysophosphatidic ethanolamine (LPE), diacylglycerol (DAG), CER, and cholesteryl ester (CE) were significantly upregulated in the plasma of patients with PTC, whereas the concentrations of FA and acylcarnitine were decreased, suggesting increased metabolism and reduced FA synthesis and β-oxidation. In addition, we applied the bubble chart to display the log_2_FC and log_10_p of different types of the differential lipids, which can better reflect the changing trend of lipids in PTC (Fig. 3C). The log_2_FC values are presented in this figure. The larger the point, the smaller the *p*-value and the more significant the difference. Similar to the scatter plot above, TAG, SM, PE, PA, LPE, DAG, CER, and CE increased, whereas phosphatidylcholine (PC), FA, and acylcarnitine decreased in the PTC group.

**Figure 4:**
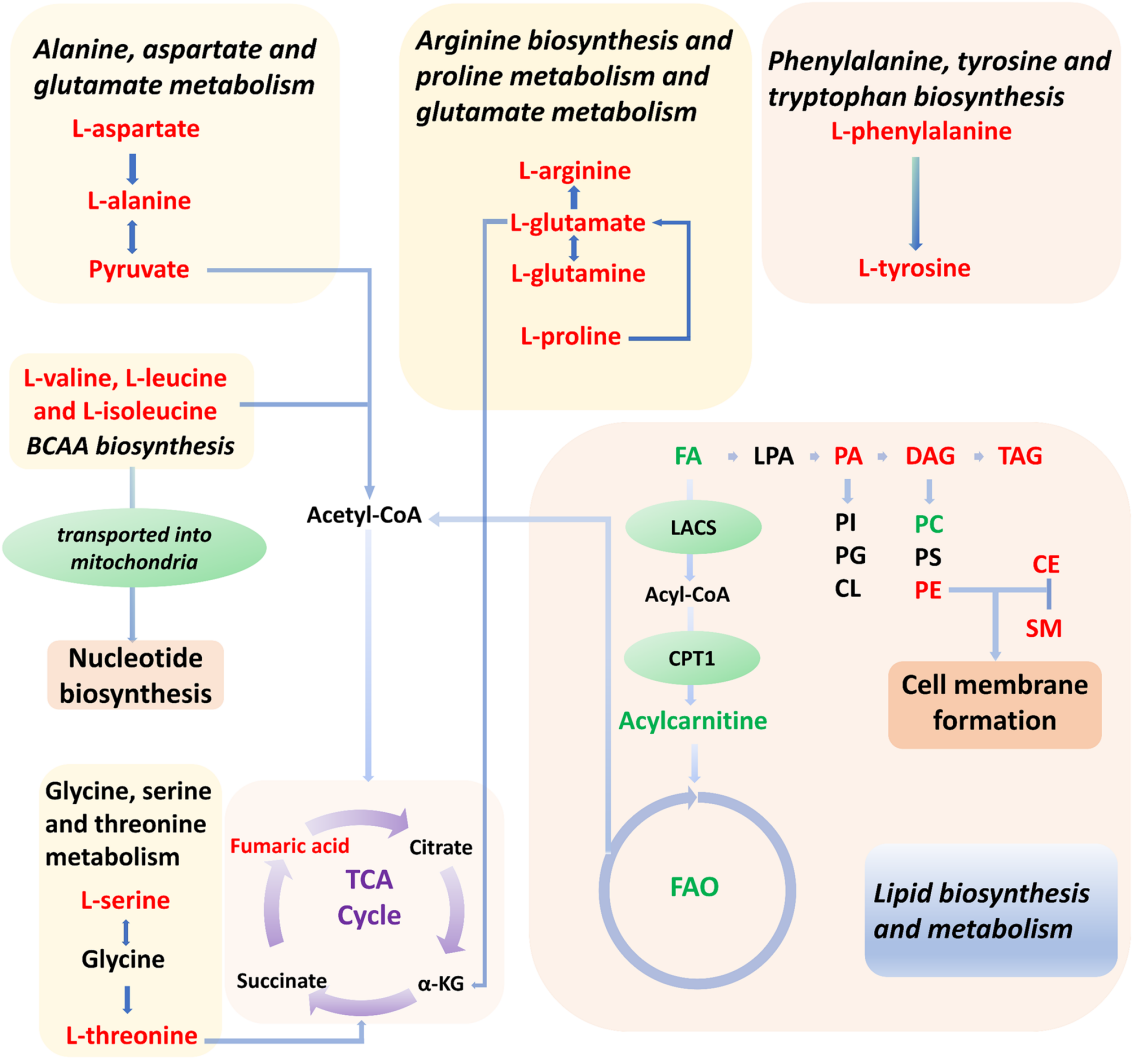
The interactions between altered metabolic pathways and lipid metabolism in PTC. Multiple metabolic and lipid reprogramming aspects reflect the potential pathogenesis of PTC. Red and green lettering of the compounds indicate elevated or lowered levels, respectively. *α-KG* α-ketoglutaric acid, *FAO* fatty acid oxidation, *CPT1* carnitine palmitoyltransferase 1, *LACS* long-chain acyl-CoA synthase, *acetyl-CoA* acetyl coenzyme A, *acyl-CoA* acyl coenzyme A, *LPA* lysophosphatidic acids, *PI* phosphatidylinositol, *PG* phosphatidylglycerol, *CL* cardiolipin, *PS* phosphatidylserine.

Based on our findings, changes in metabolic pathways and metabolic characteristics of PTC are summarized in Fig. 4. Collectively, multiple metabolic pathways, such as BCAAs, L-glutamate, L-glutamine, TCA cycle, and lipid metabolism, were substantially altered in patients with PTC. The interaction of these metabolic pathways unveils metabolic reprogramming and the potential pathogenesis of PTC.

### Establishment of the diagnostic model in discovery and validation cohorts

**Figure 5:**
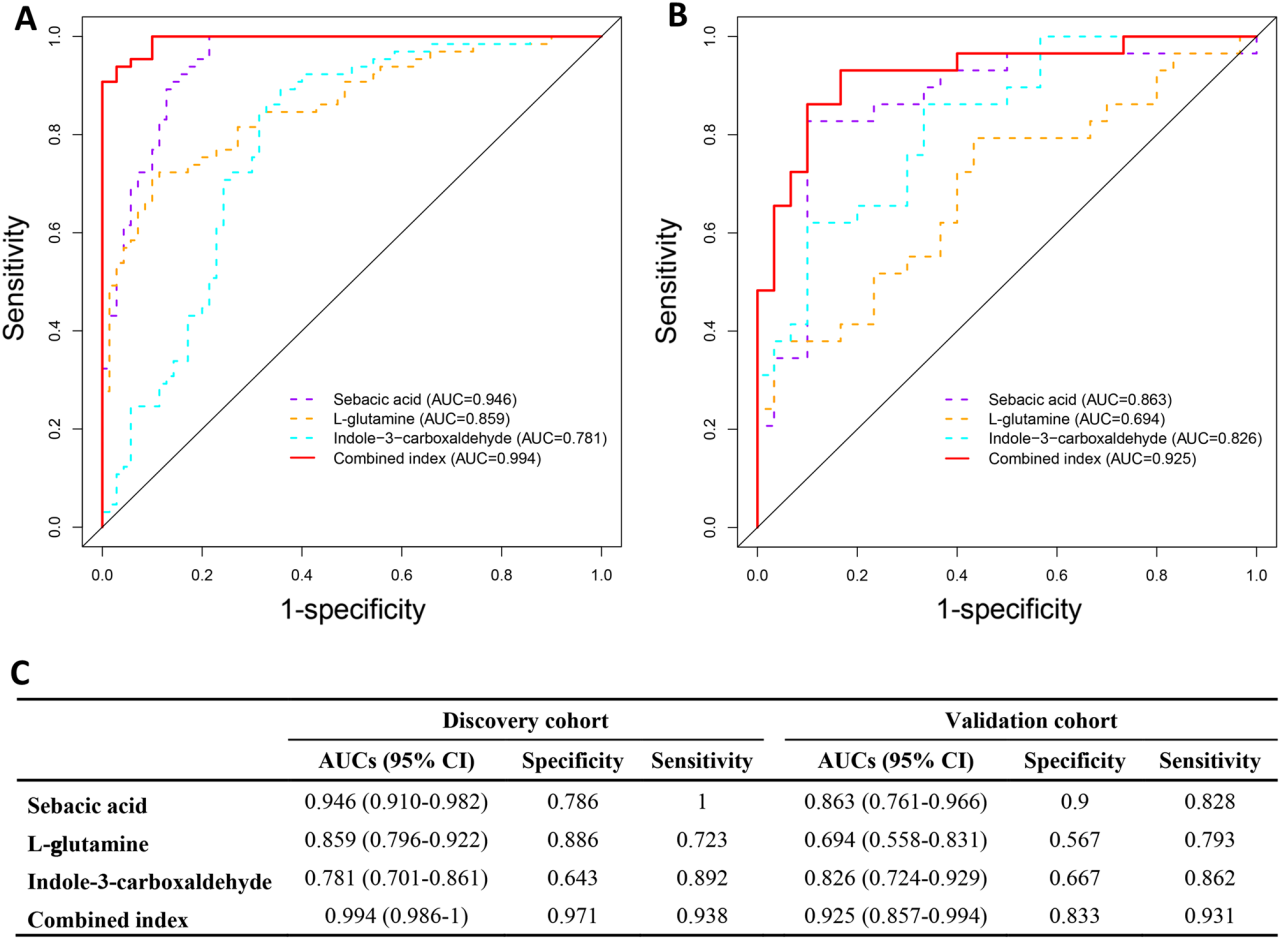
The ROC curves of the discriminative model. Three biomarkers, including sebacic acid, L-glutamine, and indole-3-carboxaldehyde, and the combined indices in the discovery (**A**) and validation cohorts (**B**). The AUC values (95% confidence intervals), 1-specificity, and sensitivity of the ROC curves in the discovery cohort and the validation cohort (**C**).

To build a diagnostic model for PTC, metabolite-based biomarkers were screened using a stepwise regression method, according to previous studies^13,14^. Differential metabolites were put into the stepwise logistic regression, and a model with a minimal Akaike information criterion value was established to identify potential biomarkers that could be used to distinguish PTC from HC. Finally, a panel of three metabolites, including sebacic acid, L-glutamine, and indole-3-carboxaldehyde, was selected to build the diagnostic model. ROC curves were used to assess the performance of a diagnostic test based on multiple classification rules, with 1-specificity on the x axis and sensitivity on the y axis. In summary, the area under the ROC curve (AUC) of individual compounds ranged from 0.781 to 0.946 in the discovery cohort, indicating that all three compounds have predictive potential for PTC. The AUC of the combined diagnosis of the three compounds was 0.994, with a sensitivity and specificity of 93.8% and 97.1%, respectively (Figs. 5A and C). The biomarkers also showed excellent diagnostic efficiency in the validation cohort, with an AUC of 0.925, sensitivity of 93.1%, and specificity of 83.3% (Fig. 5B and C). This diagnostic model showed good discrimination between PTC and HC.

## Discussion

Metabolic and lipid reprogramming plays an important role in the development of malignant tumors^15^. Circulating metabolic biomarkers support the understanding of tumor biology and early diagnosis with minimal invasion. However, the metabolic reprogramming characteristics of PTC have not been fully elucidated. Therefore, they were explored in the present study using targeted metabolomics and lipidomics.

Metabolomic and lipidomic data showed that patients with PTC have markedly different metabolic patterns compared to those of healthy subjects. Patients with PTC had higher concentrations of metabolites related to BCAAs biosynthesis, including L-threonine, L-valine, L-leucine, and L-isoleucine (Fig. 2). Metabolic reprogramming of BCAAs is related to the development of tumors. An increase in plasma BCAAs levels has also been observed in pancreatic cancer and hepatocellular carcinoma^16^. BCAAs, including L-valine, L-leucine, and L-isoleucine, can be used for protein synthesis, energy metabolism, and biosynthesis^17^. BCAAs are imported into the cell and converted into branched-chain α-keto acids (BCKAs) by branched-chain amino acid transaminases. Eventually, BCKAs are catabolized to acetyl-coenzyme A (acetyl-CoA), which enters the TCA cycle to supply energy for tumor cells. BCAAs may be beneficial to tumors by providing nitrogen for Deoxyribonucleic Acid (DNA) synthesis^18^. Elevated levels of BCAAs in the plasma and tumor tissue are often accompanied by a decrease in the breakdown of BCAAs^18^, and tumor growth may be promoted when BCAAs catabolism is inhibited^19^. The accumulation of BCAAs in tissues results in chronic activation of the mammalian target of rapamycin complex 1 (mTORC1), which is associated with cell growth and tumorigenesis^20^. A possible mechanism is that leucine binds to its sensor, Sestrin2, to activate mTORC1^20^. The activation of the mTORC1 is associated with cell growth and tumorigenesis in many human and animal models^17^. mTORC1 triggers a series of signaling pathways that regulate autophagy and lipid, nucleotide, and protein synthesis by phosphorylating its downstream effectors, including eukaryotic translation initiation factor 4E-binding protein 1, p70S6 kinase, and SREBP^21^. Therefore, elevated BCAAs levels may serve as potential targets for PTC therapy.

Pathway analysis showed that glutamate and glutamine metabolism and the TCA cycle were altered in PTC (Fig. 1). Although α-ketoglutaric acid (α-KG) and citrate were not detected, the relative concentrations of pyruvate, L-glutamate, and fumaric acid were increased (Fig. 2), indicating that TCA cycle may be upregulated in PTC. Glutamine catabolism is initiated by glutaminase (GLS) to generate Glutamate^22^. Glutamate is metabolized in the mitochondria to α-KG, which participates in the TCA cycle and provides energy for cell growth. Glutamine is a critical substrate for cytoplasmic nucleotide biosynthesis^23^. Although glutamine is considered a non-essential amino acid, most tumor cells cannot proliferate or survive in media lacking it^24^. The inhibition of GLS expression can delay tumor growth^8^. Glutathione (GSH), an antioxidant, requires glutamine to provide nitrogen and carbon sources for its synthesis. The GSH-mediated increase of antioxidant capacity may promote malignant cellular progression^25^. Ammonia, produced by glutamine catabolism, may reduce cellular sensitivity to tumor necrosis factor-α (TNF-α) through an autophagy-dependent mechanism, promoting the occurrence of tumors^26^. Because of the critical role of glutamine, the inhibition of GLS activity is a potential target for tumor therapy^8^.

Lipidomics has also been used in tumor-related studies, which have confirmed the nature of lipid changes in tumors^27^. In our study, the relative concentrations of PA, PE, CE, and SM were increased in PTC, whereas the acylcarnitine and FA contents decreased (Fig. 3A), indicating that fatty acid oxidation (FAO) was downregulated and lipid metabolism was increased in patients with PTC. FAO is an important energy source in the mitochondria. Long-chain fatty acids are converted to acyl coenzyme A (acyl-CoA) by long-chain acyl-CoA synthase. Acyl-CoA is transformed into acylcarnitine by carnitine palmitoyltransferase I. Acylcarnitine is transported into the mitochondrial matrix, and the fatty acid chain is broken. Acetyl-CoA is produced during β-oxidation and participates in the TCA cycle. FAO has different phenotypes in different tumors. FAO is upregulated in kirsten rat sarcoma viral oncogene mutant lung cancer, hepatocellular carcinoma, and triple-negative breast cancer^28^. However, excess FAO-derived Adenosine triphosphate may inhibit the survival of leukemia cells, and FAO has also been downgraded in lymphoma^29^. Moreover, FAO is activated in undifferentiated cells. FAO activation enhances the aggressiveness of ovarian cancer, making it more susceptible to metastasis^29^. Additionally, FAO is related to the degree of tumor differentiation in pediatric neuroblastoma. FAO downregulation effectively reduces tumor burden^30^. In our study, PTC showed a lower relative concentration of FA and acylcarnitine (Fig. 3A), indicating that FAO is downregulated in PTC compared to HC. As a differentiated tumor, the biological behavior and energy metabolism characteristics of PTC may be different from those of undifferentiated cancer, showing different metabolic characteristics. In summary, integrated metabolomics and lipidomics demonstrated that PTC had distinct energy metabolic reprogramming with induced BCAAs and glutamine accumulation as well as reduced FAO.

In addition, the proliferation of tumor cells requires the supply of lipids, such as PE, CE, and SM, which serve as substrates for energy metabolism, are involved in the construction of the cell membrane bilayer structure and cell signal transduction^9^. CE accumulation plays an important role in the proliferation of pancreatic cancers. CE activates PI3K/AKT signaling pathway, which promotes tumor aggressiveness^31^. Suppression of CE synthesis suppresses this signaling pathway. SM is the main component of lipid bilayer membranes, and high expression of sphingomyelin synthetase 2 (SMS2), a key enzyme in SM synthesis, is related to tumor progression. SMS2 knockout or SMS2 inhibitors can reduce tumor progression^32^. In addition, SM is associated with tumor angiogenesis^33^, potentially providing new targets for tumor treatment.

As reprogramming of metabolites may provide circulating biomarkers for cancer diagnosis, potential metabolite biomarkers for PTC were screened in this study. Finally, a panel of three metabolites, including sebacic acid, L-glutamine, and indole-3-carboxaldehyde, was selected to build the diagnostic model, achieving a very good discriminative effect in both the discovery and validation cohorts (Fig. 5). Currently, fine needle aspiration biopsy (FNAB) is the most reliable preoperative diagnostic method for PTC, but still has some limitations^34^. For example, the trauma caused by FNAB may result in histological changes that affect correct diagnosis after thyroidectomy^35^. Moreover, incomplete sampling often produces inconclusive results^36^. The introduced biomarkers for PTC such as BRAF, RAS, have poor specificity and positive predictive value^3^. Therefore, metabolite biomarkers identified in this study may provide a minimally invasive complementary approach for PTC diagnosis with high sensitivity and specificity. Furthermore, the altered metabolic pathways or related critical proteins may serve as the potential therapeutic targets, and the metabolic interventions may be exploited as new treatment strategies for PTC^7,37^.

Compared to most other studies, we enrolled a larger cohort and constructed a diagnostic model with good discrimination^34,38,39^. Jiang et al. revealed the biomarkers of PTC based on lipidomics^34^. However, without a combination of metabolomic analysis, it cannot more comprehensively explain the metabolic characteristics of PTC. Chen et al. analyzed the plasma metabolite characteristics of PTC, but their study had a small sample size^38^. Although Huang et al. had a larger sample size than ours^40^, the untargeted metabolomics method was utilized to identify the differential metabolites based on databases, with some of them being not confirmed by the standards. Moreover, mass spectrometry detector in the full scan mode in untargeted metabolomics is easily saturated, leading to the limited linear ranges, repeatability, and quantitative accuracy^41^. In our study, the standard solutions of metabolites were used to develop the targeted metabolomics and lipidomics methods using the multiple reaction monitoring mode, which has the advantages of high sensitivity, high specificity, and excellent quantification ability^42^. Nevertheless, our study has some limitations. Our research is a single-center study, which led to a lack of external verification. Therefore, a multicenter prospective study is required to validate our findings.

## Conclusions

In conclusion, we performed an integrated approach of widely targeted metabolomics and lipidomics to explore metabolic reprogramming and potential metabolite biomarkers of PTC. Distinct metabolic reprogramming was observed in PTC, especially for metabolites related to energy metabolism, such as BCAAs, L-glutamate, L-glutamine, FA, and lipids. Furthermore, we identified a panel of three circulating metabolites that may serve as diagnostic biomarkers for PTC. This panel showed excellent discrimination efficiency between PTC and HC in both the discovery and validation cohorts. Thus, it provides new targets for the comprehensive treatment of PTC.

### Supplementary Information

Supplementary Figure S1.

Supplementary Figure S2.

Supplementary Figure S3.

Supplementary Figure S4.

Supplementary Figure S5.

Supplementary Information.

## Data Availability

All datasets analyzed during the current study are not publicly available but are available from the corresponding author on reasonable request.
