# Supplementary figures and images for: Combined metabolomic and lipidomic analysis uncovers metabolic profile and biomarkers for papillary thyroid carcinoma
Source: Sci Rep. 2023 Oct 17;13:17666. doi: 10.1038/s41598-023-41176-4 (PMC10582036; doi:10.1038/s41598-023-41176-4)

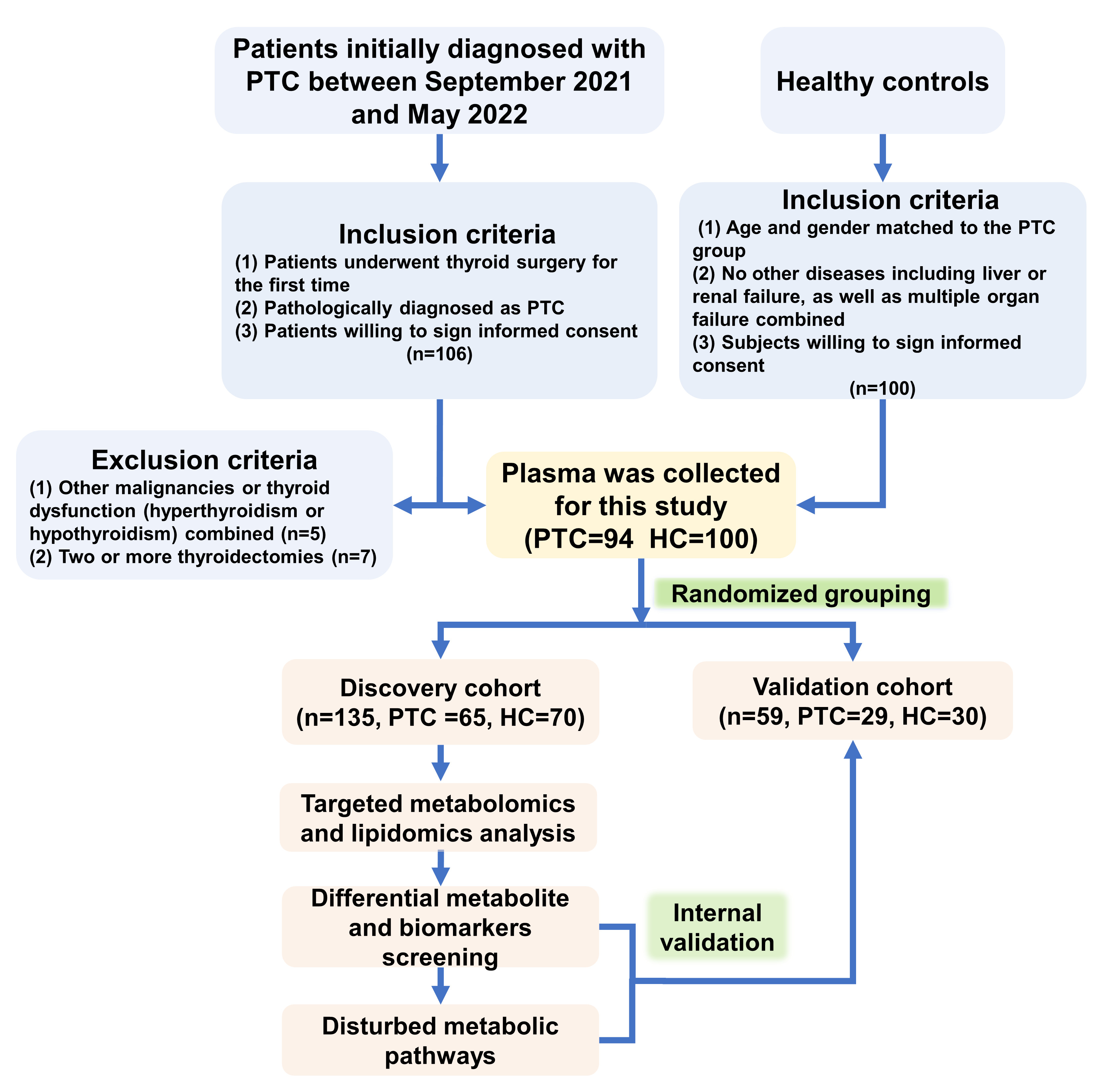

Supplement: Supplementary file 1 — Supplementary Figure S1. [file 41598_2023_41176_MOESM1_ESM.tif]

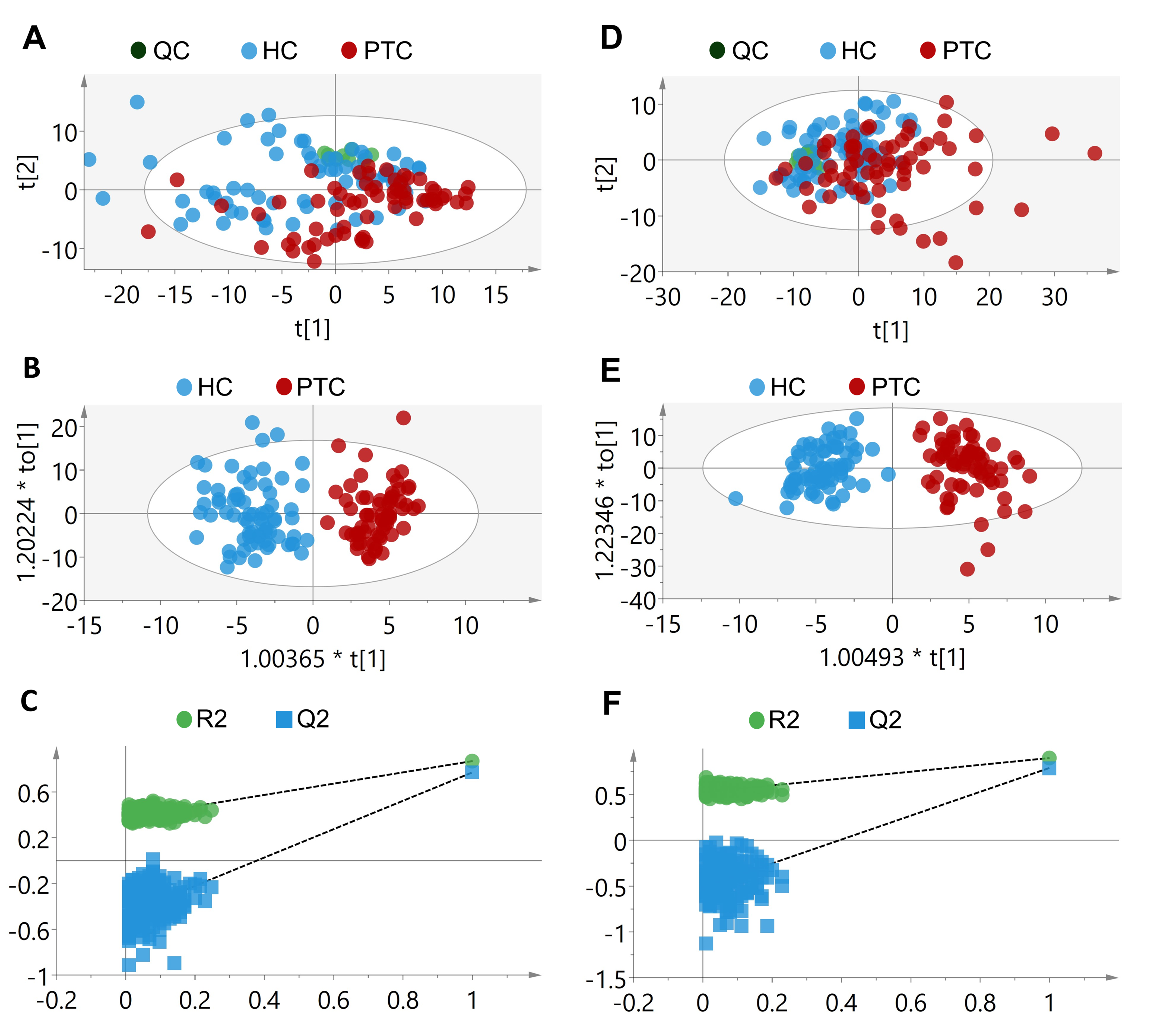

Supplement: Supplementary file 2 — Supplementary Figure S2. [file 41598_2023_41176_MOESM2_ESM.tif]

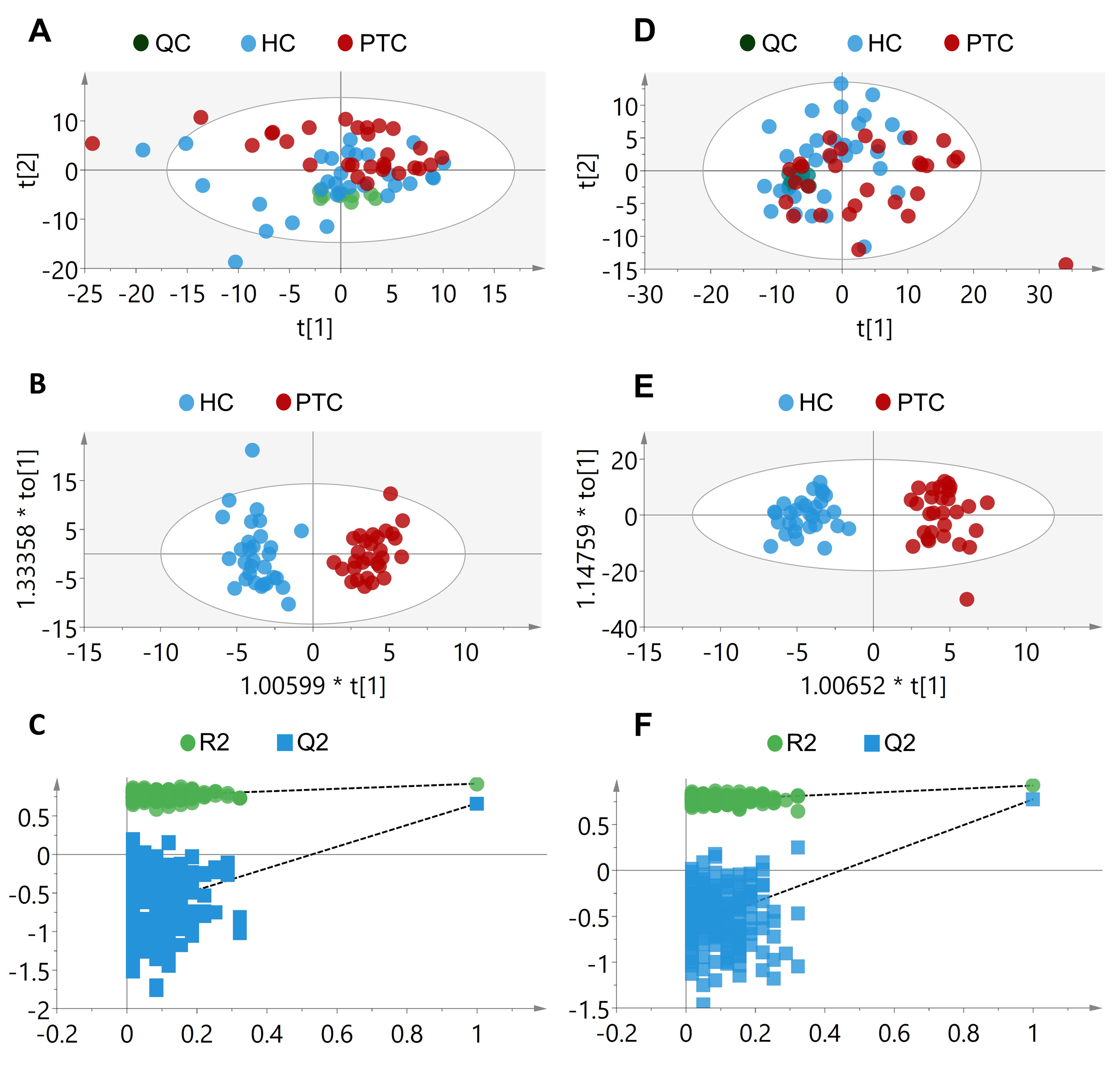

Supplement: Supplementary file 3 — Supplementary Figure S3. [file 41598_2023_41176_MOESM3_ESM.tif]

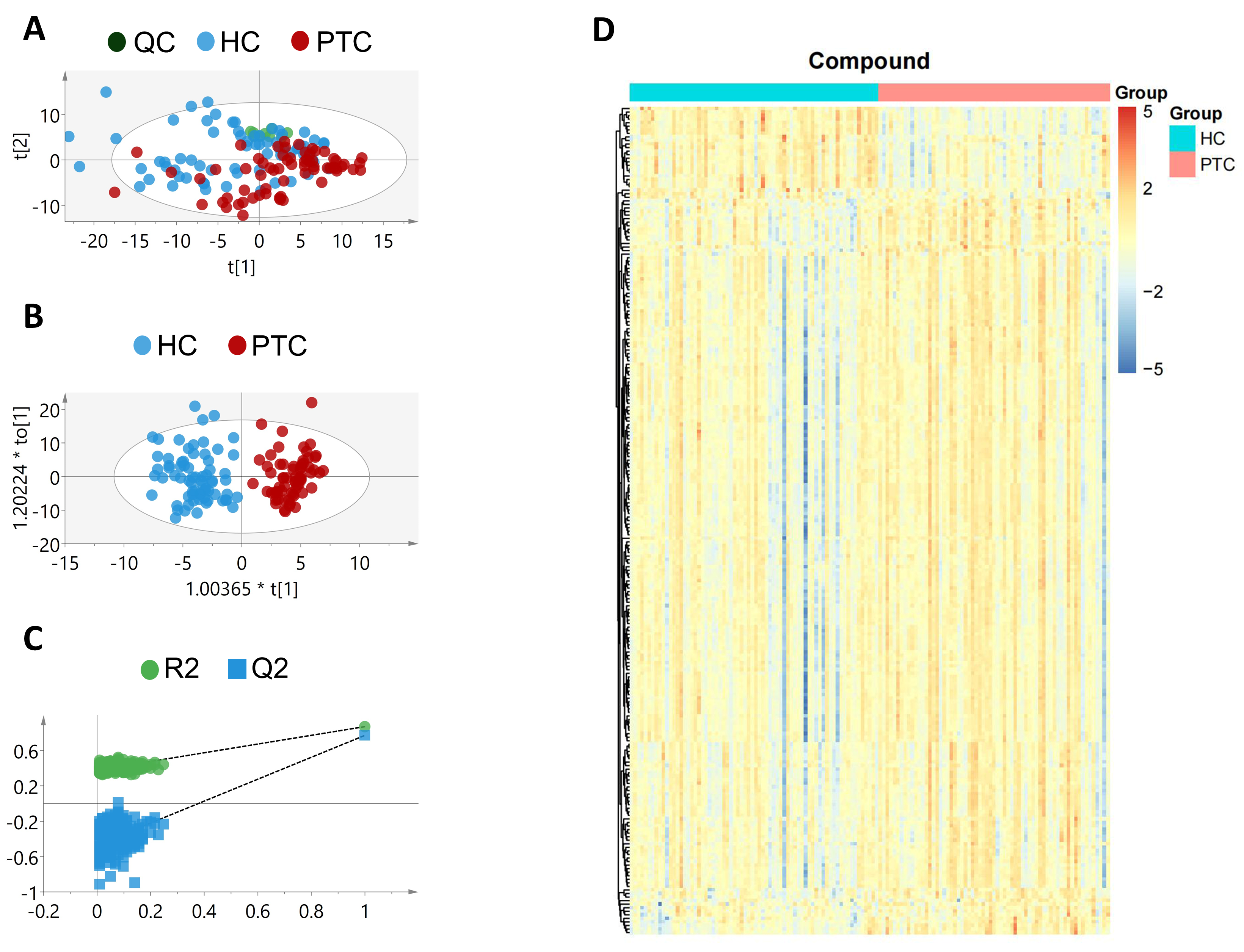

Supplement: Supplementary file 4 — Supplementary Figure S4. [file 41598_2023_41176_MOESM4_ESM.tif]

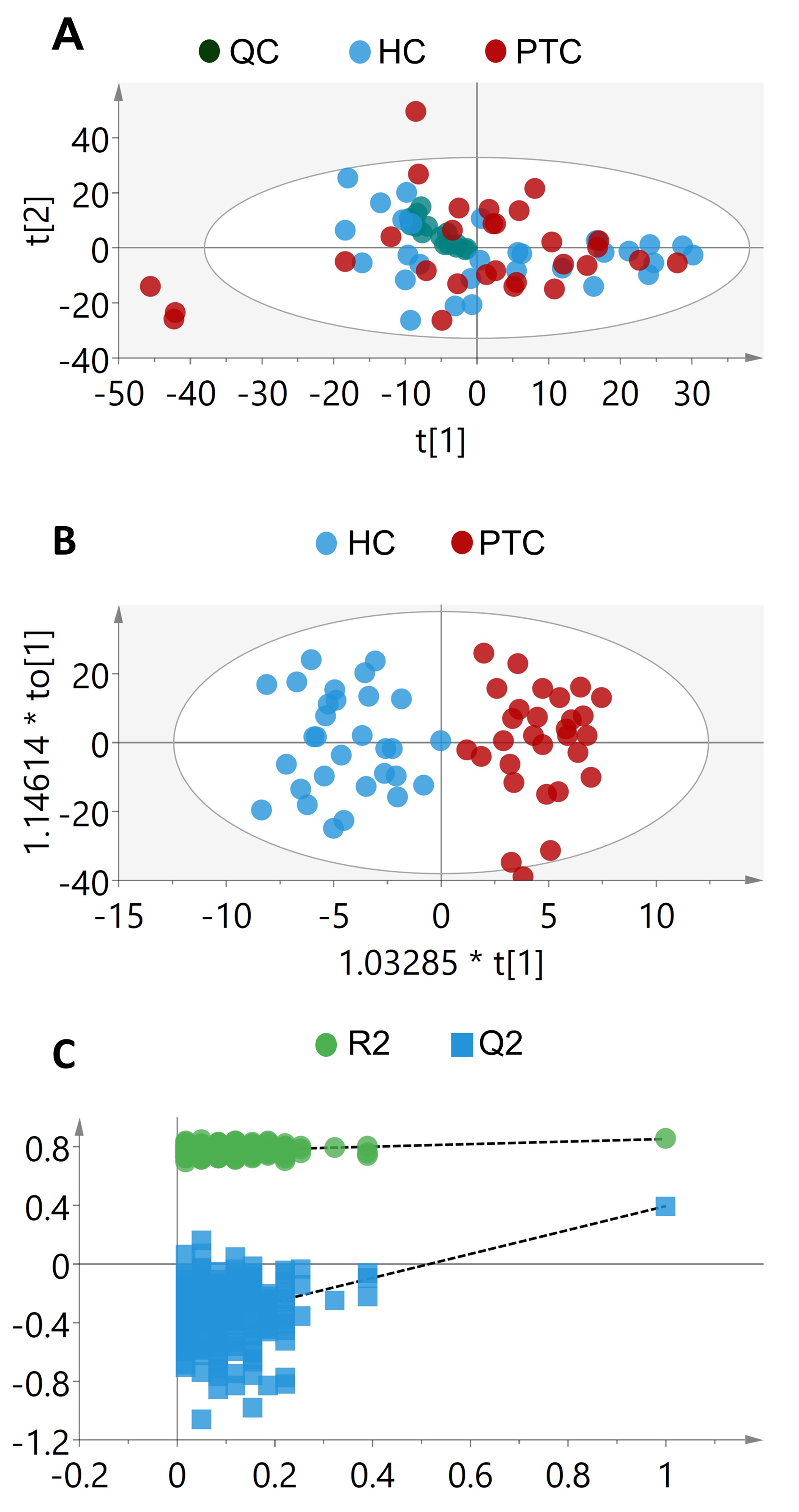

Supplement: Supplementary file 5 — Supplementary Figure S5. [file 41598_2023_41176_MOESM5_ESM.tif]
