# Supplementary material for: Combined metabolomic and lipidomic analysis uncovers metabolic profile and biomarkers for papillary thyroid carcinoma
Source: Sci Rep. 2023 Oct 17;13:17666. doi: 10.1038/s41598-023-41176-4 (PMC10582036; doi:10.1038/s41598-023-41176-4)
Supplement: Supplementary file 6 — Supplementary Information. [file 41598_2023_41176_MOESM6_ESM.docx]

**Supporting information**

**Materials and methods**

**Supporting information Figure S1-S5**

**Supporting information Table S1-S3**

**2. Materials and methods**

**2.2 Reagents and sample preparation**

Blood samples were collected from subjects using anticoagulant tubes containing EDTA-k2, centrifuged at 1500 ×*g* at 4 °C for 10 min, and the plasma was collected and stored at -80 °C.

For targeted metabolomics, 20 μL-plasma samples were mixed with 5-μL internal standard (IS (metabolomics)) and 100-μL methanol. High-performance liquid chromatography grade methanol, isopropanol, and acetonitrile were obtained from Merck KGaA (Darmstadt, Germany). Then, the mixture was shaken for 5 min, followed by twice 10-min centrifugation at 18700 ×*g* at 4 ℃. Subsequently, 70 μL of the supernatant were injected into an ultra-performance liquid chromatography-tandem mass spectrometry (UPLC-MS/MS) system. IS (metabolomics), including glycochenodeoxycholic acid-d7, cholic acid-d4, chenodeoxycholic acid-d4, trimethylamine-d9 N-oxide, indole-3-lactic acid-d5, kynurenic acid-d5, L-tryptophan-d5, succinic acid-1,4-^13^C2, L-valine-d8, 3-hydroxyanthranilic acid-d3, taurocholic acid-d5, taurochenodeoxycholic acid-d5, L-methionine-d3, and L-leucine-d3 were supplied by Toronto Research Chemicals (Ontario, Canada).

For targeted lipidomics, 10-μL internal standard (IS (lipidomics)) and 150-μL methanol were added to 20-μL plasma samples, and the mixture was shaken for 5 min. After adding 0.5 mL of tert-butyl methyl ether (MTBE), the mixture was shaken for 15 min and equilibrated at 4 °C for 10 min, followed by 10 min of centrifugation at 18700 ×*g* at 4 ℃. MTBE was obtained from Sigma-Aldrich (St. Louis, MO, USA). The supernatant (400 μL) was placed in a vacuum concentrator (Thermo Fisher, SPD2010-230) and evaporated to dryness without heating for approximately 40 min. A total of 100 μL of acetonitrile/isopropanol/water (ratio: 65:30:5) containing 5 mM ammonium acetate were added to the dried samples, and the mixture was shaken for 10 min. A Milli-Q system (Merck Millipore, Darmstadt, Germany) was used for ultrapure water purification. After twice centrifugation at 18700 ×*g* at 4 ℃ for 10 min, the supernatant was injected into the UPLC-MS/MS for lipidomic analysis. IS (lipidomics) including C15 ceramide-d7, lysophosphatidylcholine 19:0 (LPC 19:0), and palmitic acid (d31) were obtained from Avanti Polar Lipids (Alabaster, AL).

Quality control (QC) samples for plasma were prepared by taking a small volume of each experimental sample to obtain a pooled matrix sample.

**Figure legends of supporting information**

**Figure S1. Enrollment flowchart of the participants employed for screening the differential metabolites and biomarkers.** *PTC*, papillary thyroid carcinoma; *HC*, healthy controls.

**Figure S2.** **Plasma metabolome changed in patients with PTC in the discovery cohort.** Principal component analysis (PCA), orthogonal partial least squares discriminant analysis (OPLS-DA), and permutation test of plasma metabolites in patients with PTC and HC in the discovery cohort. PCA score plot (A), OPLS-DA score plot (B), and permutation test (C) on the C18 column. PCA score plot (D), OPLS-DA score (E), and permutation test (F) on the hydrophilic interaction liquid chromatography (HILIC) column. *QC,* quality control.

**Figure S3.** **The plasma metabolite profiles of patients with PTC markedly changed in the validation cohort.** PCA, OPLS-DA, and permutation test of plasma metabolites in patients with PTC and HC in the validation cohort. PCA score plot (A), OPLS-DA score plot (B), and permutation test (C) on the C18 column. PCA score plot (D), OPLS-DA score (E), and permutation test (F) on the HILIC column.

**Figure S4.** **Perturbation of PTC plasma lipids in the discovery cohort.** PCA (A), OPLS-DA (B), and permutation test (C) of plasma lipids in patients with PTC and HC on the C8 column. The cluster heat map analysis of differential lipids (D).

**Figure S5. The PTC plasma lipid profiles were significantly altered in the validation cohort.** PCA, OPLS-DA, and permutation test of plasma lipids in patients with PTC and HC in the validation cohort. PCA score plot (A), OPLS-DA score plot (B), and permutation test (C) on the C8 column.

**Table S1.** **Differential metabolites of patients with PTC versus HC.**

| Index | Metabolites | HMDB ID | p | FC | VIP |
| --- | --- | --- | --- | --- | --- |
| 1 | Cortisone | HMDB0002802 | 2.25E-05 | 0.81807 | 1.05403 |
| 2 | L-Norleucine | HMDB0001645 | 0.000152 | 1.307198 | 1.23162 |
| 3 | p-Octopamine | HMDB0004825 | 0.000417 | 1.35111 | 1.20237 |
| 4 | Norepinephrine | HMDB0000216 | 0.006232 | 1.219277 | 1.16916 |
| 5 | Indole-3-carboxaldehyde | HMDB0029737 | 6.28E-10 | 0.610749 | 1.51454 |
| 6 | Glucose 6-phosphate | HMDB0001401 | 8.96E-05 | 0.541592 | 1.09374 |
| 7 | Decanoylcarnitine | HMDB0000651 | 6.02E-07 | 0.29603 | 1.32063 |
| 8 | L-Octanoylcarnitine | HMDB0000791 | 8.59E-07 | 0.321446 | 1.29364 |
| 9 | Petroselinic acid | HMDB0002080 | 2.88E-06 | 0.373349 | 1.15391 |
| 10 | Dodecanoylcarnitine | HMDB0002250 | 1.5E-09 | 0.309086 | 1.51737 |
| 11 | Tetradecanoylcarnitine | HMDB0005066 | 9.6E-08 | 0.520853 | 1.37815 |
| 12 | 3-Hydroxyoctanoyl carnitine | HMDB0061634 | 3.54E-14 | 0.421094 | 1.80012 |
| 13 | Hydroxytetradecenoylcarnitine | HMDB0253296 | 7.18E-15 | 0.539658 | 1.76348 |
| 14 | Glutaconylcarnitine | HMDB0013129 | 2.75E-05 | 0.682258 | 1.03355 |
| 15 | 3-Hydroxy-cis-5-octenoylcarnitine | HMDB0240766 | 5.15E-06 | 0.310347 | 1.2008 |
| 16 | cis-4-Decenoyl carnitine | HMDB0240772 | 6.42E-10 | 0.403729 | 1.52845 |
| 17 | trans-2-Tetradecenoylcarnitine | HMDB0013329 | 5.78E-19 | 0.431823 | 1.98461 |
| 18 | 9-Hexadecenoylcarnitine | HMDB0013207 | 2.29E-05 | 0.663721 | 1.22537 |
| 19 | (5Z,8Z)-Tetradecadienoylcarnitine | HMDB0240756 | 1.14E-09 | 0.4327 | 1.54938 |
| 20 | Dodecadienoic acid | HMDB0302621 | 1.4E-17 | 0.485983 | 1.86809 |
| 21 | Eicosapentaenoic acid | HMDB0001999 | 4.72E-07 | 0.541005 | 1.42573 |
| 22 | Docosahexaenoic acid | HMDB0002183 | 2.15E-10 | 0.491718 | 1.67361 |
| 23 | Pentadecanoic acid | HMDB0000826 | 0.002061 | 0.810641 | 1.2061 |
| 24 | Azelaic acid | HMDB0000784 | 3.66E-09 | 0.65363 | 1.67618 |
| 25 | Suberic acid | HMDB0000893 | 3.68E-09 | 0.697817 | 1.63152 |
| 26 | Sebacic acid | HMDB0000792 | 4.57E-25 | 0.427235 | 2.22847 |
| 27 | Myristic acid | HMDB0000806 | 0.000166 | 0.722241 | 1.27326 |
| 28 | Heptadecanoic acid | HMDB0002259 | 0.018334 | 0.764817 | 1.132 |
| 29 | Stearic acid | HMDB0000827 | 0.041236 | 0.824346 | 1.10589 |
| 30 | Elaidic acid | HMDB0000573 | 0.012 | 0.793923 | 1.17552 |
| 31 | Oleic acid | HMDB0000207 | 0.017849 | 0.795353 | 1.15572 |
| 32 | Gamma-Linolenic acid | HMDB0003073 | 0.020556 | 0.792669 | 1.06774 |
| 33 | Eicosadienoic acid | HMDB0005060 | 0.001192 | 0.698847 | 1.26366 |
| 34 | Dihomo-gamma-linolenic acid | HMDB0002925 | 2.27E-06 | 0.606778 | 1.45227 |
| 35 | Cholesterol sulfate | HMDB0000653 | 0.010338 | 0.602143 | 1.00075 |
| 36 | Dodecanoic acid | HMDB0000638 | 3.28E-05 | 0.613369 | 1.12755 |
| 37 | Adrenic acid | HMDB0002226 | 3.15E-05 | 0.603216 | 1.34621 |
| 38 | Alpha-Linolenic acid | HMDB0001388 | 0.024177 | 0.797584 | 1.07489 |
| 39 | Docosapentaenoic acid | HMDB0006528 | 3.95E-07 | 0.540909 | 1.51432 |
| 40 | Arachidonic acid | HMDB0001043 | 7.53E-10 | 0.547775 | 1.67561 |
| 41 | Palmitic acid | HMDB0000220 | 0.007162 | 0.793516 | 1.22429 |
| 42 | Linoleic acid | HMDB0000673 | 0.001835 | 0.788641 | 1.2214 |
| 43 | 9-Pentadecenoic acid | HMDB0029765 | 9.51E-05 | 0.766689 | 1.17489 |
| 44 | 10Z-Heptadecenoic acid | HMDB0060038 | 0.01572 | 0.762047 | 1.15001 |
| 45 | Dihomo-alpha-linolenic acid | HMDB0060039 | 1.27E-06 | 0.608511 | 1.47048 |
| 46 | 7Z,10Z-Hexadecadienoic acid | HMDB0000477 | 2.59E-05 | 0.667471 | 1.2849 |
| 47 | Docosatrienoic acid | HMDB0002823 | 0.008768 | 0.741766 | 1.15024 |
| 48 | Tetracosatetraenoic acid (24:4n-6) | HMDB0006246 | 0.041764 | 0.716534 | 1.01611 |
| 49 | Beta-D-Fructose 6-phosphate | HMDB0003971 | 6.18E-14 | 0.334817 | 1.94045 |
| 50 | Alpha-dimorphecolic acid | HMDB0004670 | 4.79E-06 | 0.568279 | 1.20548 |
| 51 | Guanidinosuccinic acid | HMDB0003157 | 1.65E-08 | 1.5197 | 1.56809 |
| 52 | 5-Hydroxyindoleacetic acid | HMDB0000763 | 2.54E-05 | 1.644656 | 1.0786 |
| 53 | Deoxycorticosterone | HMDB0000016 | 1.73E-11 | 1.850925 | 1.41704 |
| 54 | N-Methyl-D-aspartate | HMDB0002393 | 4.35E-05 | 1.373121 | 1.30162 |
| 55 | Xanthurenic acid | HMDB0000881 | 6.48E-06 | 0.647194 | 1.20458 |
| 56 | Norvaline | HMDB0013716 | 2.25E-06 | 1.21307 | 1.33662 |
| 57 | Serotonin | HMDB0000259 | 4.55E-07 | 3.414694 | 1.37918 |
| 58 | 17-Hydroxyprogesterone | HMDB0000374 | 1.51E-11 | 1.949566 | 1.4173 |
| 59 | L-Alloisoleucine | HMDB0000557 | 2.47E-09 | 1.477269 | 1.54415 |
| 60 | L-Glutamine | HMDB0000641 | 1.7E-13 | 1.307736 | 1.77778 |
| 61 | L-Methionine | HMDB0000696 | 4.9E-07 | 1.300646 | 1.44151 |
| 62 | Creatine | HMDB0000064 | 5.25E-10 | 1.450654 | 1.52843 |
| 63 | L-Lysine | HMDB0000182 | 3.06E-06 | 1.257346 | 1.43219 |
| 64 | Glycylproline | HMDB0000721 | 0.000526 | 1.492457 | 1.01602 |
| 65 | Homocitrulline | HMDB0000679 | 0.000299 | 1.452848 | 1.15774 |
| 66 | 5-Aminolevulinic acid | HMDB0001149 | 1.57E-10 | 1.406928 | 1.66168 |
| 67 | L-Homoserine | HMDB0000719 | 7.73E-07 | 1.384835 | 1.38116 |
| 68 | L-Alanine | HMDB0000161 | 9.44E-07 | 1.34921 | 1.51179 |
| 69 | Agmatine | HMDB0001432 | 0.001234 | 1.246907 | 1.09505 |
| 70 | Citrulline | HMDB0000904 | 9.24E-05 | 1.298542 | 1.06193 |
| 71 | L-Arginine | HMDB0000517 | 0.003763 | 1.222891 | 1.09311 |
| 72 | L-Valine | HMDB0000883 | 1.1E-06 | 1.204478 | 1.36072 |
| 73 | L-Histidine | HMDB0000177 | 5.04E-09 | 1.302442 | 1.64692 |
| 74 | L-Cystine | HMDB0000192 | 1.53E-14 | 1.755 | 1.8373 |
| 75 | L-Serine | HMDB0000187 | 3.59E-08 | 1.346397 | 1.47737 |
| 76 | L-Isoleucine | HMDB0000172 | 1.33E-08 | 1.447871 | 1.48923 |
| 77 | Citicoline | HMDB0001413 | 2.22E-10 | 6.017125 | 1.6 |
| 78 | L-Proline | HMDB0000162 | 1.03E-07 | 1.332303 | 1.4708 |
| 79 | (R)-pyrrolidine-2-carboxylic acid | HMDB0003411 | 5.66E-08 | 1.326241 | 1.48805 |
| 80 | L-Leucine | HMDB0000687 | 6E-10 | 1.42392 | 1.63685 |
| 81 | Pyridoxamine | HMDB0001431 | 0.000569 | 1.353189 | 1.26937 |
| 82 | Histamine | HMDB0000870 | 3.26E-07 | 1.290935 | 1.53075 |
| 83 | Dimethylamine | HMDB0000087 | 1.67E-09 | 0.574378 | 1.43228 |
| 84 | 5-Hydroxylysine | HMDB0000450 | 3.39E-06 | 1.546308 | 1.26284 |
| 85 | L-Tyrosine | HMDB0000158 | 1.5E-07 | 1.324851 | 1.55283 |
| 86 | L-Threonine | HMDB0000167 | 5.92E-07 | 1.373188 | 1.42181 |
| 87 | p-Hydroxymandelic acid | HMDB0000822 | 0.000278 | 1.373053 | 1.28959 |
| 88 | Ribonolactone | HMDB0001900 | 1.15E-08 | 1.20405 | 1.5521 |
| 89 | Hexanoylcarnitine | HMDB0000705 | 6.24E-05 | 0.535284 | 1.13405 |
| 90 | Adipic acid | HMDB0000448 | 3.72E-13 | 1.330013 | 1.81951 |
| 91 | Aminocaproic acid | HMDB0001901 | 2.31E-08 | 1.306145 | 1.47791 |
| 92 | Adenosine monophosphate | HMDB0000045 | 3.2E-06 | 1.864425 | 1.26002 |
| 93 | Sarcosine | HMDB0000271 | 3.08E-07 | 1.363036 | 1.54137 |
| 94 | gamma-Glutamylphenylalanine | HMDB0000594 | 2.52E-06 | 1.359343 | 1.47898 |
| 95 | Glutamyltryptophan | HMDB0028830 | 0.002304 | 1.216096 | 1.17278 |
| 96 | Dodecenoylcarnitine | HMDB0251567 | 2.62E-10 | 0.467099 | 1.55075 |
| 97 | 2-trans,4-cis-Decadienoylcarnitine | HMDB0013325 | 4.91E-06 | 0.694237 | 1.24906 |
| 98 | (2E,4Z)-Decadienoylcarnitine | HMDB0240751 | 5.02E-06 | 0.692463 | 1.2384 |
| 99 | Oxidized glutathione | HMDB0003337 | 1.46E-05 | 1.931006 | 1.125 |
| 100 | Taurine | HMDB0000251 | 5.24E-08 | 1.414029 | 1.38442 |
| 101 | N-Acetyl-L-alanine | HMDB0000766 | 9.57E-08 | 1.297889 | 1.37601 |
| 102 | Pyruvate | HMDB0000243 | 2.18E-17 | 2.311365 | 1.86767 |
| 103 | Beta-Alanine | HMDB0000056 | 1.11E-08 | 1.407698 | 1.44072 |
| 104 | gamma-Glutamylalanine | HMDB0006248 | 3.63E-05 | 1.358483 | 1.22323 |
| 105 | N6-Acetyl-L-lysine | HMDB0000206 | 6.27E-06 | 1.392876 | 1.30216 |
| 106 | L-Asparate | HMDB0000191 | 9.43E-06 | 1.357396 | 1.29653 |
| 107 | L-Glutamate | HMDB0000148 | 0.000224 | 1.372775 | 1.22615 |
| 108 | L-Phenylalanine | HMDB0000159 | 3.38E-05 | 1.28482 | 1.22526 |
| 109 | Methylglutaric acid | HMDB0000752 | 3.12E-08 | 1.323886 | 1.44075 |
| 110 | Phenyllactic acid | HMDB0000779 | 0.011135 | 1.23889 | 1.08094 |
| 111 | Acetylglycine | HMDB0000532 | 1.16E-05 | 1.983147 | 1.00523 |
| 112 | Fumaric acid | HMDB0000134 | 1.66E-12 | 1.540312 | 1.61296 |

Each column of the table represents the index, the name of the differential metabolites, the Human Metabolome Database (HMDB) ID, the p-value, FC value, and VIP value of the differential metabolites. *FC,* fold change; *VIP,* variable importance in the projection.

**Table S2.** **Altered metabolic pathways in patients with PTC.**

| KEGG pathway | Total | Expected | Hits | Raw p |
| --- | --- | --- | --- | --- |
| Aminoacyl-tRNA biosynthesis | 48 | 2.5703 | 14 | 5.89E-08 |
| Biosynthesis of unsaturated fatty acids | 36 | 1.9277 | 10 | 9.15E-06 |
| Valine, leucine and isoleucine biosynthesis | 8 | 0.42839 | 4 | 0.000455 |
| Arginine biosynthesis | 14 | 0.74968 | 4 | 0.005071 |
| Glycine, serine and threonine metabolism | 33 | 1.7671 | 6 | 0.006786 |
| Arginine and proline metabolism | 38 | 2.0348 | 6 | 0.013641 |
| Alanine, aspartate and glutamate metabolism | 28 | 1.4994 | 5 | 0.014463 |
| Phenylalanine, tyrosine and tryptophan biosynthesis | 4 | 0.21419 | 2 | 0.015845 |
| Glutamine and glutamate metabolism | 6 | 0.32129 | 2 | 0.036923 |
| Cysteine and methionine metabolism | 33 | 1.7671 | 4 | 0.095909 |
| Phenylalanine metabolism | 10 | 0.53548 | 2 | 0.096421 |
| Tyrosine metabolism | 42 | 2.249 | 4 | 0.18386 |
| Citrate cycle (TCA cycle) | 20 | 1.071 | 2 | 0.29101 |
| beta-Alanine metabolism | 21 | 1.1245 | 2 | 0.3113 |
| Pyruvate metabolism | 22 | 1.1781 | 2 | 0.33148 |

*KEGG,* Kyoto Encyclopedia of Genes and Genomes.

**Table S3.** **Differential lipids of patients with PTC and HC.**

| Index | Lipids | Class | p | FC | VIP |
| --- | --- | --- | --- | --- | --- |
| 1 | CE(16:1) | CE | 0.007107 | 1.45738 | 1.18808 |
| 2 | CE(20:4) | CE | 0.000656 | 1.642659 | 1.16553 |
| 3 | CER(24:1) | CER | 0.000165 | 1.588522 | 1.48476 |
| 4 | DCER(16:0) | CER | 0.00811 | 1.567168 | 1.12362 |
| 5 | DCER(18:0) | CER | 7.12E-05 | 1.654099 | 1.57017 |
| 6 | DCER(24:1) | CER | 0.000491 | 1.499459 | 1.45153 |
| 7 | HCER(16:0) | CER | 0.00033 | 1.367791 | 1.23795 |
| 8 | HCER(20:1) | CER | 0.002106 | 1.678555 | 1.27571 |
| 9 | HCER(24:1) | CER | 0.002872 | 1.456084 | 1.29296 |
| 10 | HexCer d18:1/12:0 | CER | 0.000144 | 1.430924 | 1.42996 |
| 11 | LacCer d18:1/12:0 | CER | 0.00906 | 1.320238 | 1.21609 |
| 12 | LCER(24:1) | CER | 0.00028 | 1.535193 | 1.17137 |
| 13 | DAG(16:0/18:1) | DAG | 0.043835 | 1.386431 | 1.01536 |
| 14 | DAG(18:2/20:4) | DAG | 0.012724 | 1.38437 | 1.0576 |
| 15 | SM(16:0) | SM | 0.004112 | 1.246584 | 1.26307 |
| 16 | SM(18:0) | SM | 0.000463 | 1.35875 | 1.45564 |
| 17 | SM(18:1) | SM | 0.001279 | 1.310156 | 1.37145 |
| 18 | SM(20:1) | SM | 0.001242 | 1.314527 | 1.37052 |
| 19 | SM(22:1) | SM | 0.012186 | 1.218191 | 1.17327 |
| 20 | SM(24:1) | SM | 0.002839 | 1.261171 | 1.2861 |
| 21 | TAG40:0-FA14:0 | TAG | 0.027243 | 0.39732 | 1.00415 |
| 22 | TAG50:0-FA16:0 | TAG | 0.017659 | 1.363009 | 1.32052 |
| 23 | TAG50:0-FA18:0 | TAG | 0.006676 | 1.411124 | 1.40402 |
| 24 | TAG50:1-FA16:0 | TAG | 0.004206 | 1.351235 | 1.44119 |
| 25 | TAG50:1-FA18:1 | TAG | 0.003821 | 1.378419 | 1.44526 |
| 26 | TAG50:2-FA16:0 | TAG | 0.001441 | 1.33756 | 1.47374 |
| 27 | TAG50:2-FA16:1 | TAG | 0.020486 | 1.29888 | 1.25509 |
| 28 | TAG50:2-FA18:1 | TAG | 0.029601 | 1.247189 | 1.20692 |
| 29 | TAG50:2-FA18:2 | TAG | 0.000911 | 1.391935 | 1.52521 |
| 30 | TAG50:3-FA14:0 | TAG | 0.046267 | 1.238341 | 1.16726 |
| 31 | TAG50:3-FA16:0 | TAG | 0.0035 | 1.341298 | 1.38399 |
| 32 | TAG50:3-FA16:1 | TAG | 0.0116 | 1.297043 | 1.23101 |
| 33 | TAG50:3-FA18:1 | TAG | 0.02647 | 1.262732 | 1.20646 |
| 34 | TAG50:3-FA18:2 | TAG | 0.006141 | 1.288763 | 1.32639 |
| 35 | TAG50:3-FA18:3 | TAG | 0.012085 | 1.361214 | 1.36393 |
| 36 | TAG50:4-FA14:0 | TAG | 0.031921 | 1.310662 | 1.10601 |
| 37 | TAG50:4-FA16:0 | TAG | 0.007279 | 1.36013 | 1.30702 |
| 38 | TAG50:4-FA16:1 | TAG | 0.007652 | 1.356441 | 1.1829 |
| 39 | TAG50:4-FA18:1 | TAG | 0.014473 | 1.342995 | 1.23387 |
| 40 | TAG50:4-FA18:2 | TAG | 0.021315 | 1.314987 | 1.14595 |
| 41 | TAG50:4-FA18:3 | TAG | 0.013412 | 1.350551 | 1.28609 |
| 42 | TAG50:5-FA16:1 | TAG | 0.005004 | 1.417146 | 1.19666 |
| 43 | TAG50:5-FA18:2 | TAG | 0.013991 | 1.394555 | 1.15322 |
| 44 | TAG51:1-FA18:0 | TAG | 0.025303 | 1.390337 | 1.27273 |
| 45 | TAG51:3-FA18:2 | TAG | 0.027749 | 1.23382 | 1.1981 |
| 46 | TAG51:4-FA16:1 | TAG | 0.014193 | 1.286803 | 1.12758 |
| 47 | TAG51:4-FA18:2 | TAG | 0.011509 | 1.281473 | 1.24121 |
| 48 | TAG51:4-FA18:3 | TAG | 0.020002 | 1.263092 | 1.28076 |
| 49 | TAG51:5-FA18:2 | TAG | 0.013375 | 1.357484 | 1.13194 |
| 50 | TAG51:5-FA18:3 | TAG | 0.013594 | 1.349932 | 1.17879 |
| 51 | TAG52:0-FA16:0 | TAG | 0.007868 | 1.519849 | 1.36081 |
| 52 | TAG52:0-FA18:0 | TAG | 0.012971 | 1.558182 | 1.27657 |
| 53 | TAG52:1-FA16:0 | TAG | 0.007359 | 1.548772 | 1.37442 |
| 54 | TAG52:1-FA18:0 | TAG | 0.006681 | 1.54337 | 1.38072 |
| 55 | TAG52:1-FA20:1 | TAG | 0.010939 | 1.519075 | 1.27651 |
| 56 | TAG52:2-FA14:0 | TAG | 0.048839 | 1.317355 | 1.13718 |
| 57 | TAG52:2-FA16:0 | TAG | 0.001179 | 1.320654 | 1.48415 |
| 58 | TAG52:2-FA16:1 | TAG | 0.013472 | 1.319525 | 1.25863 |
| 59 | TAG52:2-FA18:0 | TAG | 0.000531 | 1.476425 | 1.53518 |
| 60 | TAG52:2-FA18:1 | TAG | 0.002083 | 1.28035 | 1.4016 |
| 61 | TAG52:2-FA20:0 | TAG | 0.015335 | 1.292059 | 1.30555 |
| 62 | TAG52:2-FA20:2 | TAG | 0.012791 | 1.396507 | 1.26771 |
| 63 | TAG52:3-FA16:0 | TAG | 0.000716 | 1.307131 | 1.45718 |
| 64 | TAG52:3-FA16:1 | TAG | 0.003531 | 1.338419 | 1.31861 |
| 65 | TAG52:3-FA18:0 | TAG | 0.000441 | 1.43039 | 1.5753 |
| 66 | TAG52:3-FA18:1 | TAG | 0.000431 | 1.316729 | 1.47972 |
| 67 | TAG52:3-FA18:2 | TAG | 0.000488 | 1.310729 | 1.4824 |
| 68 | TAG52:3-FA20:0 | TAG | 0.000521 | 1.374563 | 1.50599 |
| 69 | TAG52:3-FA20:1 | TAG | 0.010171 | 1.304365 | 1.26849 |
| 70 | TAG52:3-FA20:2 | TAG | 0.01149 | 1.299485 | 1.20982 |
| 71 | TAG52:4-FA16:0 | TAG | 0.000299 | 1.32908 | 1.53175 |
| 72 | TAG52:4-FA16:1 | TAG | 0.001463 | 1.329662 | 1.33316 |
| 73 | TAG52:4-FA18:1 | TAG | 0.000619 | 1.341329 | 1.4761 |
| 74 | TAG52:4-FA18:2 | TAG | 0.00076 | 1.295568 | 1.44454 |
| 75 | TAG52:4-FA18:3 | TAG | 0.002691 | 1.330209 | 1.45947 |
| 76 | TAG52:4-FA20:0 | TAG | 0.000102 | 1.420742 | 1.59787 |
| 77 | TAG52:4-FA20:2 | TAG | 0.005647 | 1.318042 | 1.28296 |
| 78 | TAG52:4-FA22:4 | TAG | 0.014004 | 1.286354 | 1.26094 |
| 79 | TAG52:5-FA16:0 | TAG | 0.000549 | 1.451492 | 1.55638 |
| 80 | TAG52:5-FA16:1 | TAG | 0.00075 | 1.370867 | 1.34119 |
| 81 | TAG52:5-FA18:1 | TAG | 0.0005 | 1.414414 | 1.45767 |
| 82 | TAG52:5-FA18:2 | TAG | 0.000155 | 1.388028 | 1.53138 |
| 83 | TAG52:5-FA18:3 | TAG | 0.001338 | 1.373549 | 1.46501 |
| 84 | TAG52:5-FA22:5 | TAG | 0.008549 | 1.312021 | 1.31252 |
| 85 | TAG52:6-FA16:0 | TAG | 0.009695 | 1.443403 | 1.28027 |
| 86 | TAG52:6-FA16:1 | TAG | 0.00045 | 1.520563 | 1.39185 |
| 87 | TAG52:6-FA18:1 | TAG | 0.001569 | 1.445407 | 1.32131 |
| 88 | TAG52:6-FA18:2 | TAG | 0.001379 | 1.463064 | 1.28369 |
| 89 | TAG52:6-FA18:3 | TAG | 0.001034 | 1.499026 | 1.37351 |
| 90 | TAG53:1-FA16:0 | TAG | 0.044295 | 1.331432 | 1.16543 |
| 91 | TAG53:1-FA17:0 | TAG | 0.020128 | 1.33815 | 1.26105 |
| 92 | TAG53:1-FA18:0 | TAG | 0.016251 | 1.330933 | 1.3094 |
| 93 | TAG53:1-FA18:1 | TAG | 0.021228 | 1.341058 | 1.27558 |
| 94 | TAG53:2-FA16:0 | TAG | 0.003031 | 1.449622 | 1.44135 |
| 95 | TAG53:2-FA17:0 | TAG | 0.017845 | 1.353839 | 1.26557 |
| 96 | TAG53:2-FA18:1 | TAG | 0.017692 | 1.339236 | 1.30107 |
| 97 | TAG53:2-FA18:2 | TAG | 0.007938 | 1.449599 | 1.33987 |
| 98 | TAG53:3-FA16:0 | TAG | 0.001768 | 1.357686 | 1.46327 |
| 99 | TAG53:3-FA17:0 | TAG | 0.012212 | 1.290153 | 1.2924 |
| 100 | TAG53:3-FA18:2 | TAG | 0.023137 | 1.253435 | 1.24376 |
| 101 | TAG53:4-FA16:0 | TAG | 0.004056 | 1.328425 | 1.42731 |
| 102 | TAG53:4-FA17:0 | TAG | 0.030097 | 1.262577 | 1.15973 |
| 103 | TAG53:4-FA18:2 | TAG | 0.002474 | 1.344057 | 1.30466 |
| 104 | TAG53:4-FA18:3 | TAG | 0.018718 | 1.27606 | 1.30996 |
| 105 | TAG53:6-FA20:4 | TAG | 0.022452 | 1.269094 | 1.05304 |
| 106 | TAG54:0-FA18:0 | TAG | 0.023451 | 1.598745 | 1.12866 |
| 107 | TAG54:1-FA16:0 | TAG | 0.038353 | 1.398688 | 1.15606 |
| 108 | TAG54:1-FA18:0 | TAG | 0.005057 | 1.514215 | 1.3872 |
| 109 | TAG54:1-FA18:1 | TAG | 0.008196 | 1.519561 | 1.29756 |
| 110 | TAG54:1-FA20:1 | TAG | 0.026691 | 1.445361 | 1.16072 |
| 111 | TAG54:2-FA16:0 | TAG | 0.040459 | 1.395328 | 1.13901 |
| 112 | TAG54:2-FA18:0 | TAG | 0.007867 | 1.520239 | 1.33189 |
| 113 | TAG54:2-FA18:1 | TAG | 0.003648 | 1.530656 | 1.39796 |
| 114 | TAG54:2-FA18:2 | TAG | 0.000582 | 1.483649 | 1.5651 |
| 115 | TAG54:2-FA20:1 | TAG | 0.03099 | 1.428584 | 1.14457 |
| 116 | TAG54:2-FA20:2 | TAG | 0.009023 | 1.32205 | 1.2523 |
| 117 | TAG54:3-FA16:0 | TAG | 0.008699 | 1.334612 | 1.24878 |
| 118 | TAG54:3-FA16:1 | TAG | 0.040259 | 1.348413 | 1.02881 |
| 119 | TAG54:3-FA18:0 | TAG | 0.00096 | 1.415263 | 1.51406 |
| 120 | TAG54:3-FA18:1 | TAG | 0.000335 | 1.374799 | 1.52862 |
| 121 | TAG54:3-FA18:2 | TAG | 0.000733 | 1.429435 | 1.55698 |
| 122 | TAG54:3-FA20:1 | TAG | 0.005078 | 1.398727 | 1.28932 |
| 123 | TAG54:3-FA20:2 | TAG | 0.005998 | 1.350298 | 1.27222 |
| 124 | TAG54:4-FA16:0 | TAG | 0.029977 | 1.23848 | 1.19342 |
| 125 | TAG54:4-FA16:1 | TAG | 0.016628 | 1.293759 | 1.05846 |
| 126 | TAG54:4-FA18:0 | TAG | 0.000193 | 1.472722 | 1.58057 |
| 127 | TAG54:4-FA18:1 | TAG | 0.000231 | 1.369319 | 1.49546 |
| 128 | TAG54:4-FA18:2 | TAG | 4.55E-05 | 1.413777 | 1.61739 |
| 129 | TAG54:4-FA20:1 | TAG | 0.029947 | 1.327323 | 1.04005 |
| 130 | TAG54:4-FA20:2 | TAG | 0.020975 | 1.245116 | 1.18564 |
| 131 | TAG54:4-FA20:4 | TAG | 0.008353 | 1.330628 | 1.23883 |
| 132 | TAG54:5-FA16:1 | TAG | 0.037511 | 1.226503 | 1.03944 |
| 133 | TAG54:5-FA18:0 | TAG | 0.00042 | 1.477494 | 1.56811 |
| 134 | TAG54:5-FA18:1 | TAG | 3.57E-05 | 1.431724 | 1.61957 |
| 135 | TAG54:5-FA18:2 | TAG | 0.000115 | 1.377165 | 1.55405 |
| 136 | TAG54:5-FA18:3 | TAG | 0.000439 | 1.439843 | 1.53534 |
| 137 | TAG54:5-FA20:2 | TAG | 0.001 | 1.36127 | 1.35105 |
| 138 | TAG54:5-FA20:3 | TAG | 0.040219 | 1.215359 | 1.15356 |
| 139 | TAG54:6-FA16:1 | TAG | 0.01958 | 1.250474 | 1.09124 |
| 140 | TAG54:6-FA18:1 | TAG | 0.000139 | 1.548544 | 1.53791 |
| 141 | TAG54:6-FA18:2 | TAG | 2.73E-05 | 1.470341 | 1.59422 |
| 142 | TAG54:6-FA18:3 | TAG | 0.000418 | 1.484845 | 1.46615 |
| 143 | TAG54:6-FA20:3 | TAG | 0.009984 | 1.281879 | 1.1759 |
| 144 | TAG54:7-FA16:1 | TAG | 0.026568 | 1.266527 | 1.07656 |
| 145 | TAG54:7-FA18:1 | TAG | 0.001582 | 1.573919 | 1.35117 |
| 146 | TAG54:7-FA18:2 | TAG | 0.000852 | 1.555406 | 1.31238 |
| 147 | TAG54:7-FA18:3 | TAG | 0.001273 | 1.561253 | 1.30875 |
| 148 | TAG54:7-FA20:4 | TAG | 0.001526 | 1.402448 | 1.33594 |
| 149 | TAG54:8-FA18:2 | TAG | 0.017837 | 1.504621 | 1.06846 |
| 150 | TAG54:8-FA18:3 | TAG | 0.023936 | 1.50255 | 1.03033 |
| 151 | TAG54:8-FA20:4 | TAG | 0.011704 | 1.376161 | 1.09841 |
| 152 | TAG54:8-FA20:5 | TAG | 0.041221 | 1.318215 | 1.02774 |
| 153 | TAG55:2-FA18:2 | TAG | 0.01506 | 1.403455 | 1.24411 |
| 154 | TAG55:3-FA18:1 | TAG | 0.001392 | 1.497035 | 1.44026 |
| 155 | TAG55:3-FA18:2 | TAG | 0.005459 | 1.442357 | 1.35606 |
| 156 | TAG55:4-FA18:1 | TAG | 0.000232 | 1.480062 | 1.48399 |
| 157 | TAG55:4-FA18:2 | TAG | 0.000162 | 1.478448 | 1.49034 |
| 158 | TAG55:5-FA18:1 | TAG | 0.00537 | 1.298388 | 1.29564 |
| 159 | TAG55:5-FA18:2 | TAG | 3.35E-05 | 1.525916 | 1.55427 |
| 160 | TAG56:10-FA18:2 | TAG | 0.049253 | 1.332079 | 1.04903 |
| 161 | TAG56:3-FA18:0 | TAG | 0.022024 | 1.46477 | 1.17761 |
| 162 | TAG56:3-FA20:2 | TAG | 0.018473 | 1.431023 | 1.22429 |
| 163 | TAG56:4-FA18:1 | TAG | 0.024227 | 1.354818 | 1.07849 |
| 164 | TAG56:4-FA20:2 | TAG | 0.005037 | 1.384837 | 1.23768 |
| 165 | TAG56:4-FA22:4 | TAG | 0.029369 | 1.284869 | 1.14662 |
| 166 | TAG56:5-FA16:0 | TAG | 0.032917 | 1.283467 | 1.11733 |
| 167 | TAG56:5-FA18:1 | TAG | 0.002562 | 1.350398 | 1.31679 |
| 168 | TAG56:5-FA18:2 | TAG | 0.001712 | 1.435964 | 1.28901 |
| 169 | TAG56:5-FA20:2 | TAG | 0.00017 | 1.473027 | 1.35195 |
| 170 | TAG56:5-FA20:3 | TAG | 0.025058 | 1.274097 | 1.13809 |
| 171 | TAG56:5-FA22:4 | TAG | 0.0159 | 1.314466 | 1.16814 |
| 172 | TAG56:6-FA16:0 | TAG | 0.027414 | 1.239685 | 1.16833 |
| 173 | TAG56:6-FA18:0 | TAG | 0.033937 | 1.233059 | 1.15572 |
| 174 | TAG56:6-FA18:1 | TAG | 0.01119 | 1.274785 | 1.19795 |
| 175 | TAG56:6-FA18:3 | TAG | 0.003381 | 1.402428 | 1.28308 |
| 176 | TAG56:6-FA20:2 | TAG | 6.27E-05 | 1.524439 | 1.40204 |
| 177 | TAG56:6-FA20:3 | TAG | 0.003547 | 1.361769 | 1.1639 |
| 178 | TAG56:6-FA20:4 | TAG | 0.006202 | 1.30322 | 1.22208 |
| 179 | TAG56:6-FA22:4 | TAG | 0.012456 | 1.294231 | 1.24621 |
| 180 | TAG56:7-FA16:1 | TAG | 0.027439 | 1.245675 | 1.08003 |
| 181 | TAG56:7-FA18:1 | TAG | 0.049195 | 1.209801 | 1.04844 |
| 182 | TAG56:7-FA18:2 | TAG | 0.001799 | 1.341811 | 1.27669 |
| 183 | TAG56:7-FA18:3 | TAG | 0.006375 | 1.329213 | 1.2473 |
| 184 | TAG56:7-FA20:3 | TAG | 0.001378 | 1.419722 | 1.17784 |
| 185 | TAG56:7-FA20:4 | TAG | 0.001667 | 1.356423 | 1.25299 |
| 186 | TAG56:7-FA22:4 | TAG | 0.004517 | 1.320193 | 1.29311 |
| 187 | TAG56:8-FA18:1 | TAG | 0.023137 | 1.270372 | 1.166 |
| 188 | TAG56:8-FA18:2 | TAG | 0.01039 | 1.29442 | 1.12878 |
| 189 | TAG56:8-FA20:4 | TAG | 0.000443 | 1.474354 | 1.2919 |
| 190 | TAG56:8-FA20:5 | TAG | 0.011244 | 1.318395 | 1.1924 |
| 191 | TAG56:9-FA20:4 | TAG | 0.005181 | 1.439916 | 1.16031 |
| 192 | TAG56:9-FA20:5 | TAG | 0.0131 | 1.404124 | 1.12025 |
| 193 | TAG58:6-FA18:1 | TAG | 0.0309 | 1.277255 | 1.09467 |
| 194 | TAG58:6-FA22:4 | TAG | 0.005608 | 1.358628 | 1.27487 |
| 195 | TAG58:7-FA18:2 | TAG | 0.001733 | 1.355062 | 1.28612 |
| 196 | TAG58:7-FA22:4 | TAG | 0.000597 | 1.385375 | 1.27058 |
| 197 | Decanoylcarnitine | Acylcarnitine | 5.45E-07 | 0.35021 | 2.11316 |
| 198 | Lauroylcarnitine | Acylcarnitine | 1.68E-11 | 0.384712 | 2.46796 |
| 199 | Myristoylcarnitine | Acylcarnitine | 1.75E-07 | 0.621639 | 2.08288 |
| 200 | Octanoylcarnitine | Acylcarnitine | 8.53E-06 | 0.381042 | 1.96046 |
| 201 | Decenoyl-carnitine | Acylcarnitine | 3.81E-10 | 0.530892 | 2.32065 |
| 202 | 3-Hydroxy-decenoyl- carnitine | Acylcarnitine | 7.81E-06 | 0.357501 | 1.3953 |
| 203 | Dodecenoyl-carnitine | Acylcarnitine | 1.08E-11 | 0.502815 | 2.39956 |
| 204 | Tetradecenoyl-carnitine | Acylcarnitine | 2.02E-08 | 0.528675 | 2.16951 |
| 205 | Palmitoleoyl- carnitine | Acylcarnitine | 0.01306 | 0.814399 | 1.39218 |
| 206 | (5Z,8Z)-Tetradecadienoylcarnitine | Acylcarnitine | 5.73E-10 | 0.524249 | 2.29482 |
| 207 | PA(18:0/20:1) | PA | 0.011064 | 1.262464 | 1.05996 |
| 208 | PA(18:0/22:4) | PA | 2.78E-06 | 1.467032 | 1.60641 |
| 209 | LPC(14:0) +AcO | LPC | 0.003693 | 0.809358 | 1.24583 |
| 210 | LPC(18:2) +AcO | LPC | 0.000129 | 1.268163 | 1.26802 |
| 211 | PC(14:0/14:0)+AcO | PC | 0.009131 | 0.711632 | 1.21771 |
| 212 | LPE(18:2) | LPE | 0.0002 | 1.363127 | 1.16857 |
| 213 | LPE(18:3) | LPE | 0.000164 | 1.431763 | 1.27797 |
| 214 | PE(O-18:0/20:1) | PE | 0.001334 | 1.297068 | 1.02956 |
| 215 | PE(O-18:0/22:4) | PE | 0.00089 | 1.353415 | 1.31729 |
| 216 | PE(P-16:0/20:1) | PE | 0.005879 | 1.256522 | 1.16244 |
| 217 | PE(P-16:0/22:4) | PE | 1.06E-05 | 1.491636 | 1.50278 |
| 218 | PE(P-18:0/22:4) | PE | 7.49E-08 | 2.025564 | 1.83613 |
| 219 | PE(P-18:1/20:1) | PE | 0.011129 | 1.333263 | 1.02589 |
| 220 | PE(P-18:1/22:4) | PE | 0.000232 | 1.273766 | 1.25813 |
| 221 | 5_Dodecenoic acid | FA | 2.57E-06 | 0.466105 | 1.92842 |
| 222 | myristate | FA | 0.006878 | 0.8056 | 1.1545 |
| 223 | myristoleate | FA | 0.000432 | 0.601844 | 1.6248 |
| 224 | Myristelaidic acid | FA | 0.000516 | 0.605245 | 1.61894 |
| 225 | heptadecanoate | FA | 0.01828 | 0.818748 | 1.05442 |
| 226 | Nonadeca_10Z_enoic acid | FA | 0.009757 | 0.759942 | 1.27075 |
| 227 | cis-11,14-Eicosadienoic acid ester | FA | 0.007367 | 0.756802 | 1.37397 |
| 228 | cis-8,11,14-Eicosatrienoic acid ester | FA | 3.38E-05 | 0.679299 | 1.72313 |
| 229 | arachidonate | FA | 5.59E-08 | 0.58903 | 1.95505 |
| 230 | cis-11,14,17-Eicosatrienoic acid ester | FA | 2.72E-05 | 0.675741 | 1.73101 |
| 231 | cis-5,8,11,14,17-Eicosapentaenoic acid ester | FA | 1.02E-06 | 0.558167 | 1.70078 |
| 232 | docosatetraenoate | FA | 0.000108 | 0.680772 | `1.58939 |
| 233 | docosapentaenoate | FA | 2.46E-05 | 0.591715 | 1.68549 |
| 234 | cis-4,7,10,13,16,19-Docosahexaenoic acid ester | FA | 6.29E-09 | 0.553363 | 2.12115 |
| 235 | Docosapentaenoic acid (22n-6) | FA | 1.89E-05 | 0.587673 | 1.69609 |
| 236 | Docosatrienoic acid | FA | 0.026869 | 0.820393 | 1.19678 |

Each column of the table represents the index, the name of the differential lipids, the lipid class, the p-value, FC value, and VIP value of the differential lipids. *CE,* cholesteryl ester; *CER,* ceramide; *DAG,* diacylglycerol; *SM,* sphingomyelin; *TAG,* triacylglyceride; *PA,* phosphatidic acid; *LPC,* lysophosphalipid; *PC,* phosphatidylcholine; *LPE,* lysophosphatidic ethanolamine; *PE,* phosphatidyl ethanolamine; and *FA,* fatty acid.
